# Association between the C-reactive protein–triglyceride–glucose index and incident cardiovascular disease in middle-aged and older adults with arthritis: a nationwide prospective cohort study with hospital-based cross-sectional replication

**DOI:** 10.3389/fimmu.2026.1854928

**Published:** 2026-06-10

**Authors:** Feng Luo, Jia-jie Guo, Ting Xu, Xue-mei Yuan, Jia-kun Gao, Xi-cheng Zhang, Chen Peng, Qiu-yi Wang, Yi Ling, Xue-ming Yao, Wu-kai Ma

**Affiliations:** 1 Guizhou University of Traditional Chinese Medicine, Guiyang, China; 2 Department of Rheumatology and Immunology, The Second Affiliated Hospital of Guizhou University of Traditional Chinese Medicine, Guiyang, China; 3 The Second School of Clinical Medicine, Guangzhou Medical University, Guangzhou, China; 4 School of Nursing, Nanjing Medical University, Nanjing, China; 5 Department of Cardiology, Quanzhou First Hospital Affiliated to Fujian Medical University, Quanzhou, China; 6 Nanshan College, Guangzhou Medical University, Guangzhou, China

**Keywords:** arthritis, cardiovascular disease, C-reactive protein-triglyceride-glucose index, cross-sectional replication, cumulative exposure, two-time-point level-change patterns

## Abstract

**Background:**

Arthritis encompasses heterogeneous joint and rheumatic conditions and is associated with increased cardiovascular disease (CVD) risk. The C-reactive protein–triglyceride–glucose index (CTI) integrates inflammatory and metabolic burden, but its association with CVD among adults with arthritis remains unclear.

**Methods:**

Data from the China Health and Retirement Longitudinal Study (CHARLS) and an independent hospital-based cross-sectional replication cohort were analyzed. In the CHARLS, baseline CTI was assessed in 2,894 adults with self-reported physician-diagnosed arthritis or rheumatism. Cumulative CTI exposure (cumCTI) and exploratory two-time-point CTI level-change patterns were evaluated in 1,394 participants using Wave 3 in 2015 as the time origin. Cox regression, restricted cubic spline, subgroup, sensitivity, and exploratory predictive performance analyses were performed for incident CVD. In the hospital-based cohort, 7,960 participants were analyzed using logistic regression and spline analyses for prevalent CVD.

**Results:**

In the CHARLS, 771 incident CVD events occurred in the baseline CTI analysis, and 300 occurred in the repeated-measures analyses. In fully adjusted models, baseline CTI was associated with incident CVD [hazard ratio (HR) = 1.251, 95% confidence interval (CI): 1.083–1.446, *p* = 0.002], with the highest risk in Q4 versus Q1 (HR = 1.587, 95% CI: 1.257–2.003; *p* for trend < 0.001). cumCTI was also associated with incident CVD (HR = 1.118, 95% CI: 1.025–1.220, *p* = 0.012), with higher risk in Q4 versus Q1 (HR = 1.504, 95% CI: 1.038–2.179; *p* for trend = 0.012). Compared with Cluster 1, both Cluster 2 and Cluster 3 were associated with higher CVD risk. In the hospital-based cross-sectional replication cohort, baseline CTI was associated with prevalent CVD recorded in hospital medical records. Exploratory predictive analyses showed modest discrimination for CTI [area under the curve (AUC) = 0.556, 95% CI: 0.529–0.583], and adding CTI to the basic model changed Harrell’s C-index from 0.615 to 0.618.

**Conclusion:**

Higher CTI-related inflammatory-metabolic burden was associated with increased CVD risk in middle-aged and older adults with arthritis. CTI may provide complementary information for recognizing inflammatory-metabolic cardiovascular vulnerability in adults with arthritis, but its incremental predictive value was modest and requires further validation.

## Graphical Abstract

**Figure:**
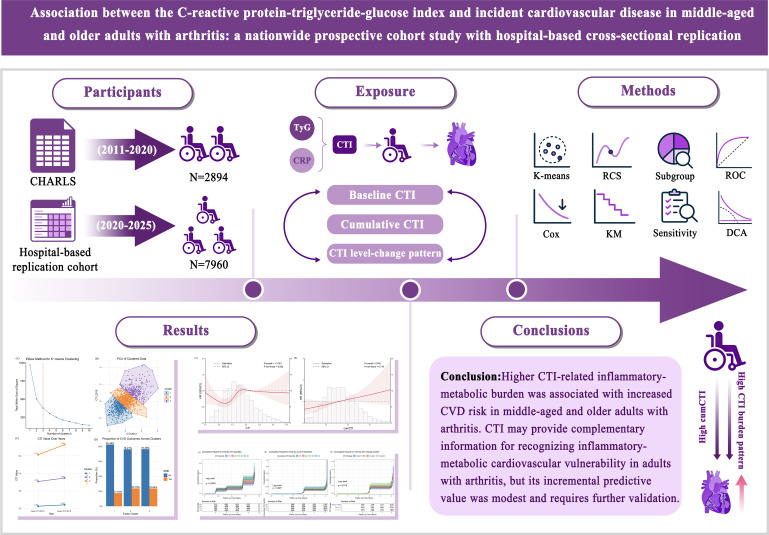

## 1 Introduction

Arthritis, a spectrum of conditions primarily involving joint inflammation and structural damage, poses a substantial global health burden (1). Osteoarthritis (OA) and rheumatoid arthritis (RA) represent the two most common forms of this disorder.

Estimates from the Global Burden of Disease (GBD) findings indicate that OA constituted approximately 7.6% of the world’s population in 2020, whereas RA affected approximately 17.9 million people in 2021 (2, 3). The significance of this burden is acutely felt in China, where a 2014 national survey reported an overall arthritis prevalence of 31.4% (4). In China, the age-standardized incidence rate for RA rose from 11.6 to 13.7 per 100,000 (1990–2021), with higher rates in women, and a Bayesian model projects a further rise to roughly 16.4 per 100,000 by 2046 (5). In light of rapid demographic aging underway in China and many other developing nations, the future burden of arthritis is poised to escalate considerably, highlighting the critical importance of advancing preventive and therapeutic measures (6).

Beyond its direct articular consequences, arthritis shows an association with increased cardiovascular disease (CVD) susceptibility (7). Chronic systemic inflammation serves as a key pathophysiological link between these conditions (8). Inflammatory mediators not only contribute to joint damage but also accelerate atherosclerosis, endothelial dysfunction, and plaque instability, thereby driving CVD pathogenesis (9). Consequently, individuals with arthritis represent a high-risk population for CVD, and conventional risk factor management, such as controlling hypertension and dyslipidemia, often fails to fully mitigate this risk, leaving a significant residual burden (10). This residual risk may be partly attributable to the interplay of chronic inflammation and impaired metabolic homeostasis, which are not sufficiently addressed by conventional cardiovascular risk management.

In this context, the concurrent evaluation of inflammation and insulin resistance (IR) has emerged as a potential approach to understanding residual CVD risk in clinically heterogeneous arthritis populations (11). C-reactive protein (CRP) reflects systemic inflammation, whereas the triglyceride–glucose (TyG) index serves as a surrogate marker of IR. Elevated CRP and TyG levels have both been associated with increased CVD risk (12, 13). Building on these two pathways, prior studies in general and hypertensive populations have suggested that the C-reactive protein–triglyceride–glucose index (CTI), an integrated inflammatory-metabolic marker, is associated with higher risks of incident CVD and stroke (14, 15). However, existing evidence on CTI has largely relied on cross-sectional or single time-point assessments, which may not fully capture cumulative exposure or short-term changes in metabolic-inflammatory burden (16). This limitation may be particularly relevant in adults with arthritis, who often experience persistent inflammatory burden and a higher prevalence of metabolic comorbidities (17). Therefore, the associations of baseline CTI, cumulative CTI exposure (cumCTI), and exploratory two-time-point CTI level-change patterns with incident CVD among individuals with arthritis remain unclear.

We therefore used the China Health and Retirement Longitudinal Study (CHARLS) to evaluate the associations of baseline CTI, time-weighted cumCTI, and exploratory two-time-point CTI level-change patterns with incident CVD in middle-aged and older adults with arthritis. To examine whether the baseline CTI–CVD association could also be observed in an independent clinical setting, we additionally assessed the cross-sectional association between baseline CTI and prevalent CVD recorded in hospital medical records in a hospital-based cross-sectional replication cohort.

## 2 Methods

### 2.1 Study population and participants

The CHARLS is an ongoing nationally representative longitudinal survey of Chinese adults aged ≥45 years, based on multistage stratified probability-proportionate-to-size sampling. In Wave 1 (2011–2012), 17,708 participants from 150 counties/districts and 450 villages in 28 provinces were enrolled. Standardized questionnaires and biomarker measurements were collected at baseline, and participants were observed through subsequent waves in 2013, 2015, 2018, and 2020 (18). The study was approved by the Institutional Review Board of Peking University (IRB00001052-11015), and all participants provided written informed consent.

**Figure 1:**
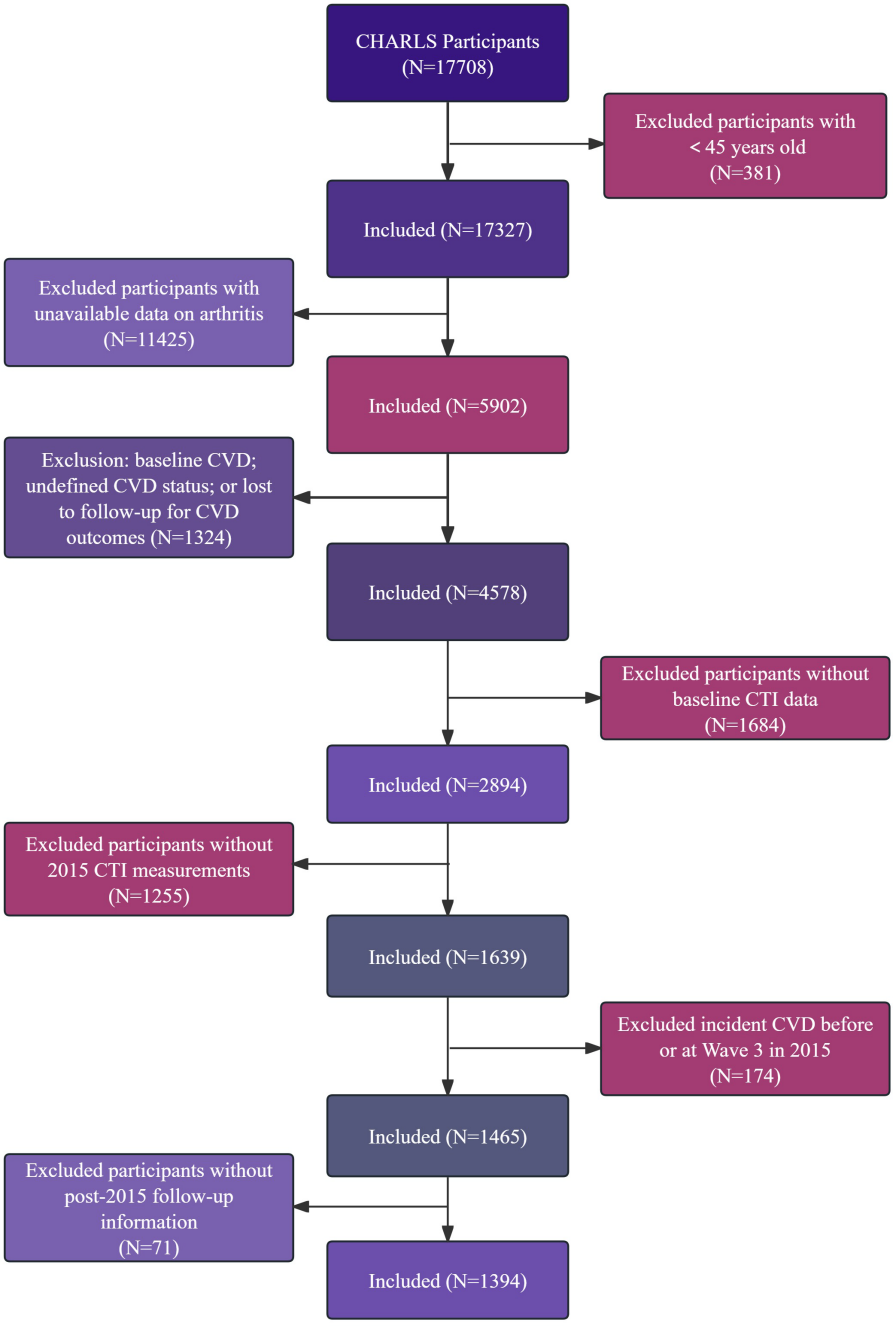
Flowchart of the study population in CHARLS. CTI, C-reactive protein–triglyceride–glucose index; CVD, cardiovascular disease; CHARLS, China Health and Retirement Longitudinal Study.

For the CHARLS analyses, 381 participants aged <45 years were first excluded from the 17,708 baseline participants, leaving 17,327 eligible individuals. After restricting the sample to participants with self-reported physician-diagnosed arthritis or rheumatism, 5,902 adults remained. A total of 1,324 participants with prevalent CVD at baseline, undefined CVD status, or missing follow-up CVD information required for the baseline CTI analysis were further excluded, leaving 4,578 participants. After exclusion of 1,684 individuals without baseline CTI data, 2,894 participants were included in the baseline CTI analysis. A further 1,255 participants without 2015 CTI measurements were excluded, leaving 1,639 participants with repeated CTI measurements in 2012 and 2015. Because cumCTI and CTI change-pattern exposures were constructed using both 2012 and 2015 CTI values, Wave 3 in 2015 was used as the time origin for these repeated-measures exposure analyses. Therefore, 174 participants who developed CVD before or at Wave 3 in 2015, including incident CVD events reported during the 2013 and 2015 follow-up waves, were excluded. An additional 71 participants without post-2015 follow-up information were further excluded. Finally, 1,394 participants were included in the cumCTI and CTI change-pattern analyses (**Figure 1**).

For the hospital-based cross-sectional replication cohort, patients aged ≥45 years with arthritis or rheumatic diseases documented in hospital medical records at the Second Affiliated Hospital of Guizhou University of Traditional Chinese Medicine between January 2020 and December 2025 were included. As shown in **Supplementary Figure 1**, 18,752 patients were screened. After excluding 4,034 individuals aged <45 years, 14,718 remained eligible. Furthermore, 4,132 patients with missing data required for CTI calculation and 2,626 patients with missing covariate data were excluded. Consequently, 7,960 patients were included in the cross-sectional replication analysis. The study was approved by the Ethics Committee of the Second Affiliated Hospital of Guizhou University of Traditional Chinese Medicine (LW20250827), and all participants provided written informed consent.

### 2.2 Assessment of CTI and cumCTI

CTI and cumCTI were calculated as follows:


CTI=0.412×ln(CRP [mg/L])+ln(TG [mg/dL]×FBG [mg/dL])/2

(19).


$$\text{cumCTI}=\frac{{\text{CTI}}^{2012}+{\text{CTI}}^{2015}}{2}\times \text{time interval}\;(2015–2012)$$

(20–22).

A linear model was applied to determine the cumCTI, which was derived by multiplying the average CTI value across the 2012 and 2015 surveys by the time interval between these two measurements. CTI_2012_ and CTI_2015_ represent the CTI values obtained during Wave 1 (2012) and Wave 3 (2015), respectively, separated by a 3-year period. This methodology follows the approach previously established for cumulative exposure indices within the CHARLS cohort and other epidemiological studies, where cumulative burden was quantified as the product of the mean biomarker level and the time interval between repeated measurements (21). The calculation code is provided in Text S1. To explore inter-individual heterogeneity in CTI burden across two biomarker waves, K-means clustering was applied using CTI measurements from baseline and 2015. Given that only two CTI measurements were available, the resulting clusters were interpreted as exploratory two-time-point CTI level-change patterns rather than complex longitudinal trajectories.

### 2.3 Assessment of arthritis and incident CVD

Arthritis status in the CHARLS was defined according to the self-reported response to the question: “Have you been diagnosed with arthritis or rheumatism by a doctor?” Participants who answered “yes” were classified as having self-reported physician-diagnosed arthritis or rheumatism. Because this item combines arthritis and rheumatism in a single question, the CHARLS cohort could not distinguish specific arthritis subtypes or broader rheumatic conditions (23). In the hospital-based cross-sectional replication cohort, arthritis or rheumatic disease status was determined from hospital medical records rather than self-report. In the CHARLS, heart disease was defined as self-reported physician-diagnosed heart attack, angina pectoris, coronary heart disease (CHD), congestive heart failure, or other heart disease, and stroke was also defined according to self-reported physician diagnosis. CVD was defined as self-reported physician-diagnosed heart disease and/or stroke, consistent with the operational definition commonly used in CHARLS-based epidemiological studies. For the prospective analysis, follow-up time was calculated from baseline to the first occurrence of CVD, death, loss to follow-up, or the end of follow-up, whichever came first. In the hospital-based cross-sectional replication cohort, prevalent CVD was identified from hospital medical records rather than self-report. This outcome was analyzed cross-sectionally and was used for cross-sectional replication of the association between baseline CTI and prevalent CVD, rather than for prognostic validation of future CVD events.

### 2.4 Covariates

This study selected covariates based on clinical significance and previous literature (24, 25). The covariates considered in this analysis comprised sociodemographic factors (sex, geographic region, marital status, education level, residence place, and age), lifestyle-related factors (smoking status, drinking status, sleep duration, and participation in social activities), and metabolic or clinical factors, including diabetes, cancer, chronic lung disease (CLD), memory-related disease, digestive system disease (liver disease and stomach disease), estimated glomerular filtration rate (eGFR) and remnant cholesterol (RC). Detailed data on these covariates are available in Text S2.

### 2.5 Statistical analysis

All analyses were performed using R 4.4.2. A two-sided *p* < 0.05 was considered statistically significant, and the Benjamini–Hochberg procedure was applied to control the false discovery rate. Continuous variables were presented as mean ± standard deviation or median (interquartile range), and categorical variables were presented as frequencies and percentages. Between-group differences were assessed using ANOVA or the Kruskal–Wallis test for continuous variables and the χ^2^ test for categorical variables.

Participants with missing key variables required to define eligibility, exposure, or outcome status were excluded. For CHARLS covariates with missing proportions <10% (**Supplementary Table 1**), random forest imputation was applied, whereas complete-case analysis was used in the hospital-based cross-sectional replication cohort (26, 27). To assess potential selection bias, included and excluded participants were compared across the baseline CTI cohort, the cumCTI/CTI level-change pattern cohort, and the hospital-based cohort (**Supplementary Tables 2**–**4**). Briefly, baseline CTI-related measures were generally comparable in the CHARLS analytic cohorts, although several demographic and clinical characteristics differed; in the hospital-based cross-sectional replication cohort, included participants were older and had higher inflammatory-metabolic burden.

K-means clustering was used to identify exploratory two-time-point CTI level-change patterns based on baseline and 2015 CTI values. The optimal cluster number was determined using the elbow method and visualized by principal component analysis. For baseline CTI, follow-up started at Wave 1. For cumCTI and CTI level-change pattern analyses, Wave 3 in 2015 was used as the time origin because these exposures incorporated CTI values from both Wave 1 and Wave 3; participants who developed CVD before or at Wave 3 were excluded.

Cox proportional-hazards models were used to examine the associations of baseline CTI, cumCTI, and CTI level-change patterns with incident CVD, with hazard ratios (HRs) and 95% confidence intervals (CIs) calculated. Model 1 was unadjusted; Model 2 was adjusted for age, sex, and marital status; and Model 3 was further adjusted for education level, residence place, geographic region, smoking status, drinking status, sleep duration, social activities, diabetes, cancer, CLD, memory-related disease, digestive system disease, eGFR, and RC. The low-stable CTI pattern served as the reference. Multicollinearity was assessed using variance inflation factors (**Supplementary Table 5**), and Kaplan–Meier curves with log-rank tests were used to compare cumulative CVD incidence.

Restricted cubic splines (RCSs) were used to evaluate non-linearity, and two-piecewise Cox models were applied when non-linear associations were detected. Subgroup analyses tested interactions across predefined strata. In the hospital-based cohort, multivariable logistic regression assessed the cross-sectional association between baseline CTI and prevalent CVD, with odds ratios (ORs) and 95% CIs estimated; RCS analysis was also performed. Exploratory predictive performance analyses included time-dependent receiver operating characteristic (ROC) curves, decision curve analysis (DCA), Harrell’s C-index, continuous net reclassification improvement (NRI), and integrated discrimination improvement (IDI). Sensitivity analyses involved the following: i) excluding participants who developed CVD within the first 2 years, ii) restricting to participants with complete covariate data, iii) excluding participants who died during follow-up, iv) additional adjustment for body mass index (BMI) and physical activity (PA), and v) jointly modeling baseline CTI and delta CTI.

## 3 Results

### 3.1 K-means cluster analysis of exploratory two-time-point CTI level-change patterns

**Figure 2:**
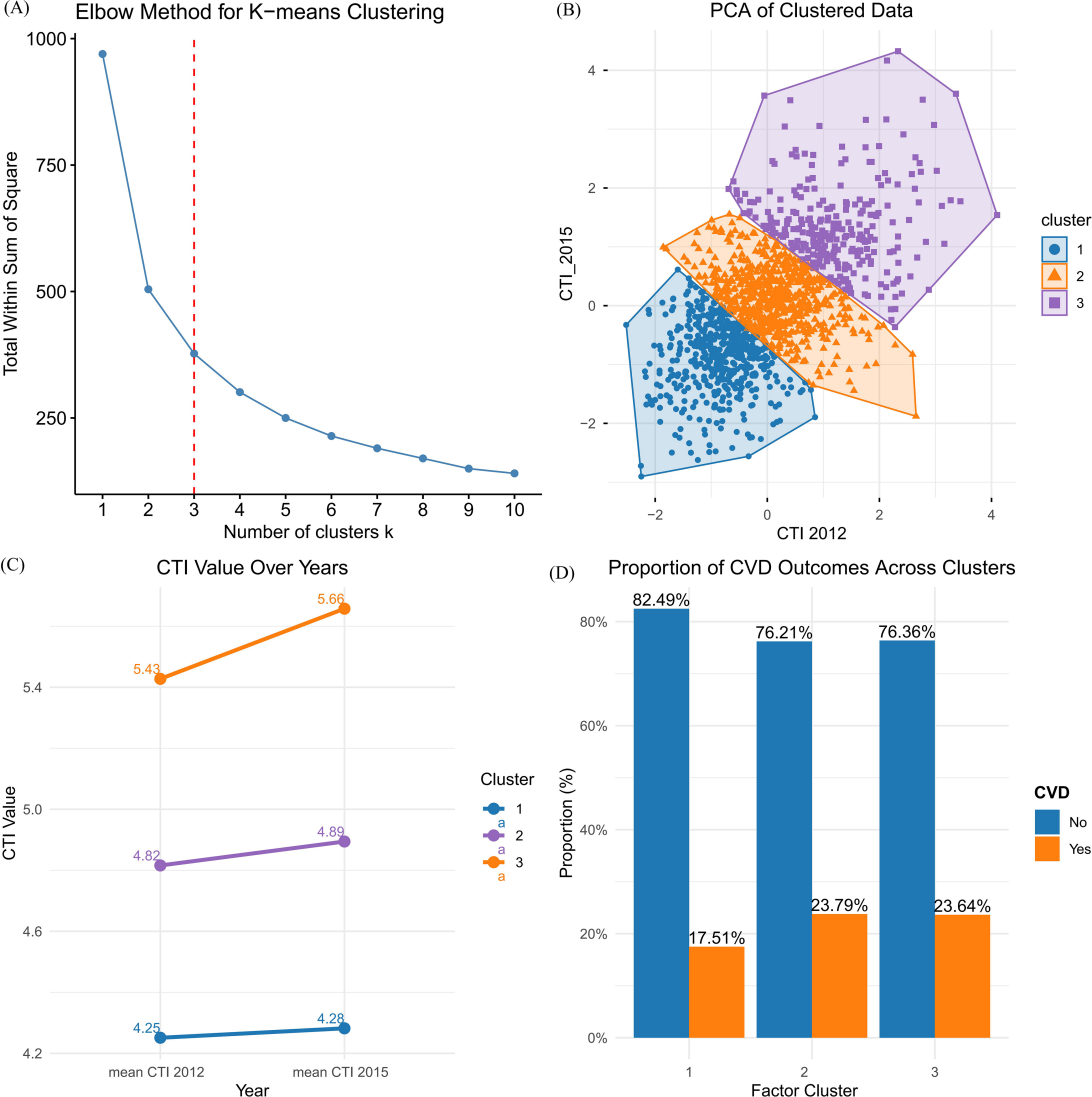
K-means clustering of CTI level-change patterns based on CTI measurements at baseline and in 2015. **(A)** Elbow method for determining the optimal number of clusters. **(B)** PCA plot of clustered data. **(C)** Mean CTI values across the two biomarker waves. **(D)** Proportion of CVD outcomes across clusters. CTI, C-reactive protein–triglyceride–glucose index; CVD, cardiovascular disease; PCA, principal component analysis.

Based on CTI measurements at baseline and in 2015, three exploratory two-time-point CTI level-change patterns were identified through K-means clustering (**Figures 2A, B**). Cluster 1 represented a low-stable pattern, Cluster 2 showed a moderate-stable/slightly increasing pattern, and Cluster 3 represented a high-increasing pattern (**Figure 2C**). The proportions of CVD were 17.51%, 23.79%, and 23.64% in Cluster 1, Cluster 2, and Cluster 3, respectively (**Figure 2D**).

### 3.2 Baseline characteristics according to baseline CTI quartiles and CTI level-change pattern clusters

**Table 1** presents the baseline characteristics of 2,894 participants in the CHARLS cohort stratified by baseline CTI quartiles. Participants in higher CTI quartiles generally showed higher CRP, fasting blood glucose (FBG), triglyceride (TG), RC, and total cholesterol (TC) levels and lower high-density lipoprotein cholesterol (HDL-C) and eGFR. The prevalence of diabetes and CVD also increased across CTI quartiles. **Table 2** summarizes the 1,394 participants included in the CTI level-change pattern analysis; Cluster 1 showed the lowest inflammatory-metabolic burden, Cluster 3 showed the highest CTI-related burden, and Cluster 2 generally showed an intermediate profile. In the hospital-based cohort, higher baseline CTI quartiles were also accompanied by less favorable inflammatory-metabolic profiles and higher prevalence of CVD (**Supplementary Table 6**).

**Table 1:** Baseline characteristics by baseline CTI quartiles.

Characteristic	Baseline characteristics by CTI quartiles						
	Overall	Q1	Q2	Q3	Q4	*p*-value^a^	*p*-adjust^b^
	(n = 2,894)	(n = 724)	(n = 723)	(n = 724)	(n = 723)		
Age, years	59	57	59	59	59	<0.001	<0.001
	(53.00, 65.00)	(51.00, 64.00)	(54.00, 66.00)	(53.00, 66.00)	(54.00, 67.00)		
Sleep duration, h	6	6	6	6	6	0.555	0.601
	(5.00, 8.00)	(5.00, 7.62)	(5.00, 8.00)	(5.00, 7.00)	(5.00, 8.00)		
eGFR, mL/min/1.73 m^2^	98.85	100.96	98.7	97.55	96.99	<0.001	<0.001
	(88.03, 105.51)	(93.40, 107.01)	(88.06, 104.80)	(85.25, 104.80)	(85.40, 105.37)		
TC, mg/dL	192.14	183.83	193.69	196.78	197.55	<0.001	<0.001
	(168.56, 218.33)	(163.92, 204.12)	(169.33, 216.88)	(173.49, 221.91)	(169.52, 230.41)		
HDL-C, mg/dL	50.64	58.76	53.35	49.1	42.14	<0.001	<0.001
	(41.75, 61.08)	(49.87, 68.81)	(45.23, 63.21)	(41.37, 57.22)	(34.41, 50.84)		
LDL-C, mg/dL	114.82	110.18	119.07	119.46	112.89	<0.001	<0.001
	(93.94, 139.18)	(91.53, 127.58)	(96.65, 142.66)	(99.26, 144.20)	(88.14, 140.72)		
RC, mg/dL	19.72	11.98	17.01	23.58	32.47	<0.001	<0.001
	(11.60, 32.09)	(6.96, 19.33)	(10.82, 25.52)	(14.69, 34.02)	(19.52, 52.38)		
Baseline CRP, mg/L	1.01 (0.56, 2.17)	0.43 (0.33, 0.59)	0.77 (0.59, 1.12)	1.36 (0.95, 2.16)	3.57 (2.04, 7.15)	<0.001	<0.001
Baseline TG, mg/dL	105.76	70.8	100	124.79	159.3	<0.001	<0.001
	(74.34, 149.57)	(57.30, 92.04)	(75.22, 130.10)	(92.93, 163.73)	(106.64, 239.39)		
Baseline FBG, mg/dL	101.88	97.47	100.44	103.5	109.62	<0.001	<0.001
	(94.50, 112.50)	(91.08, 104.76)	(93.42, 108.90)	(95.94, 113.81)	(99.36, 127.44)		
Baseline CTI	4.69 (4.35, 5.10)	4.14 (3.96, 4.24)	4.52 (4.44, 4.60)	4.88 (4.78, 4.97)	5.44 (5.24, 5.73)	<0.001	<0.001
Age group, n (%)						0.001	0.002
<60	1,534 (53.0)	432 (59.7)	366 (50.6)	367 (50.7)	369 (51.0)		
≥0	1,360 (47.0)	292 (40.3)	357 (49.4)	357 (49.3)	354 (49.0)		
Sex, n (%)						0.153	0.209
Female	1,215 (42.0)	317 (43.8)	309 (42.7)	278 (38.4)	311 (43.0)		
Male	1,679 (58.0)	407 (56.2)	414 (57.3)	446 (61.6)	412 (57.0)		
Marital status, n (%)						0.309	0.402
Unmarried	356 (12.3)	78 (10.8)	86 (11.9)	91 (12.6)	101 (14.0)		
Married	2,538 (87.7)	646 (89.2)	637 (88.1)	633 (87.4)	622 (86.0)		
Education level, n (%)						0.586	0.609
<High school	2,687 (92.8)	678 (93.6)	666 (92.1)	668 (92.3)	675 (93.4)		
≥High school	207 (7.2)	46 (6.4)	57 (7.9)	56 (7.7)	48 (6.6)		
Residence place, n (%)						<0.001	<0.001
Rural	1,988 (68.7)	534 (73.8)	508 (70.3)	482 (66.6)	464 (64.2)		
Urban	906 (31.3)	190 (26.2)	215 (29.7)	242 (33.4)	259 (35.8)		
Geographic region, n (%)						0.504	0.596
South	1,918 (66.3)	495 (68.4)	480 (66.4)	475 (65.6)	468 (64.7)		
North	976 (33.7)	229 (31.6)	243 (33.6)	249 (34.4)	255 (35.3)		
Smoking status, n (%)						0.548	0.601
Never	1,865 (64.4)	477 (65.9)	450 (62.2)	478 (66.0)	460 (63.6)		
Ever	252 (8.7)	62 (8.6)	59 (8.2)	64 (8.8)	67 (9.3)		
Now	777 (26.8)	185 (25.6)	214 (29.6)	182 (25.1)	196 (27.1)		
Drinking status, n (%)						0.007	0.011
Non-drinker	2,053 (70.9)	483 (66.7)	504 (69.7)	533 (73.6)	533 (73.7)		
Drinker	841 (29.1)	241 (33.3)	219 (30.3)	191 (26.4)	190 (26.3)		
Social activities, n (%)						0.003	0.005
No	2,438 (84.2)	618 (85.4)	634 (87.7)	586 (80.9)	600 (83.0)		
Yes	456 (15.8)	106 (14.6)	89 (12.3)	138 (19.1)	123 (17.0)		
Diabetes, n (%)						<0.001	<0.001
No	2,464 (85.1)	681 (94.1)	644 (89.1)	628 (86.7)	511 (70.7)		
Yes	430 (14.9)	43 (5.9)	79 (10.9)	96 (13.3)	212 (29.3)		
Cancer, n (%)						0.407	0.504
No	2,876 (99.4)	717 (99.0)	721 (99.7)	720 (99.4)	718 (99.3)		
Yes	18 (0.6)	7 (1.0)	2 (0.3)	4 (0.6)	5 (0.7)		
CLD, n (%)						0.065	0.094
No	2,511 (86.8)	647 (89.4)	624 (86.3)	628 (86.7)	612 (84.6)		
Yes	383 (13.2)	77 (10.6)	99 (13.7)	96 (13.3)	111 (15.4)		
Memory-related disease, n (%)						0.774	0.774
No	2,855 (98.7)	713 (98.5)	716 (99.0)	714 (98.6)	712 (98.5)		
Yes	39 (1.3)	11 (1.5)	7 (1.0)	10 (1.4)	11 (1.5)		
Digestive system disease, n (%)						0.029	0.044
No	1,909 (66.0)	447 (61.7)	477 (66.0)	487 (67.3)	498 (68.9)		
Yes	985 (34.0)	277 (38.3)	246 (34.0)	237 (32.7)	225 (31.1)		
Incident CVD during follow-up, n (%)						<0.001	<0.001
No	2,123 (73.4)	575 (79.4)	529 (73.2)	510 (70.4)	509 (70.4)		
Yes	771 (26.6)	149 (20.6)	194 (26.8)	214 (29.6)	214 (29.6)		

CTI, C-reactive protein–triglyceride–glucose index; CVD, cardiovascular disease; eGFR, estimated glomerular filtration rate; CLD, chronic lung disease; TC, total cholesterol; HDL-C, high-density lipoprotein cholesterol; LDL-C, low-density lipoprotein cholesterol; RC, remnant cholesterol; CRP, C-reactive protein; TG, triglyceride; FBG, fasting blood glucose.

^a^ *p*-Values for continuous variables calculated using ANOVA or Kruskal–Wallis test and for categorical variables using chi-squared test.

^b^ Adjusted *p*-values calculated using the Benjamini–Hochberg method.

**Table 2:** Baseline characteristics by CTI K-means clusters.

Characteristic	Baseline characteristics by CTI K-means clusters					
	Overall	Cluster 1	Cluster 2	Cluster 3	*p*-value^a^	*p*-adjust^b^
	(n = 1,394)	(n = 497)	(n = 622)	(n = 275)		
Age, years	58.00 (53.00, 64.00)	58.00 (52.00, 64.00)	58.00 (53.00, 64.00)	58.00 (53.00, 63.00)	0.705	0.731
Sleep duration, h	6.00 (5.00, 8.00)	6.00 (5.00, 8.00)	6.00 (5.00, 7.50)	6.00 (5.00, 7.00)	0.171	0.228
eGFR, mL/min/1.73 m^2^	100.20 (90.58, 105.85)	101.23 (94.87, 106.77)	99.76 (89.40, 105.65)	98.20 (85.20, 104.85)	<0.001	<0.001
TC, mg/dL	191.75 (168.17, 217.56)	184.79 (165.08, 206.44)	194.07 (168.94, 218.62)	204.90 (177.45, 236.02)	<0.001	<0.001
HDL-C, mg/dL	50.26 (41.37, 60.60)	56.83 (48.71, 66.88)	48.90 (41.75, 57.60)	40.59 (33.44, 48.71)	<0.001	<0.001
LDL-C, mg/dL	114.05 (92.78, 136.37)	110.95 (91.24, 129.12)	116.75 (95.10, 140.72)	114.05 (90.08, 140.53)	0.005	0.01
RC, mg/dL	20.49 (11.60, 33.25)	13.14 (7.73, 21.26)	22.62 (14.30, 33.63)	34.02 (23.20, 58.96)	<0.001	<0.001
Baseline CRP, mg/L	0.94 (0.53, 1.98)	0.53 (0.36, 0.80)	1.16 (0.70, 2.15)	2.42 (1.28, 4.64)	<0.001	<0.001
Baseline TG, mg/dL	107.53 (74.34, 153.99)	77.00 (61.95, 104.43)	117.26 (84.07, 156.20)	170.80 (120.80, 261.96)	<0.001	<0.001
Baseline FBG, mg/dL	102.24 (94.68, 112.45)	98.82 (91.98, 106.02)	102.96 (95.76, 112.45)	109.62 (99.36, 129.24)	<0.001	<0.001
Baseline CTI	4.68 (4.33, 5.09)	4.26 (4.07, 4.45)	4.81 (4.57, 5.05)	5.39 (5.12, 5.74)	<0.001	<0.001
Following CTI	4.79 (4.42, 5.19)	4.33 (4.06, 4.53)	4.89 (4.66, 5.10)	5.58 (5.37, 5.88)	<0.001	<0.001
cumCTI	14.21 (13.30, 15.31)	12.96 (12.44, 13.35)	14.53 (14.08, 14.99)	16.35 (15.93, 17.03)	<0.001	<0.001
Age group, n (%)					0.463	0.589
<60	777 (55.7)	270 (54.3)	345 (55.5)	162 (58.9)		
≥60	617 (44.3)	227 (45.7)	277 (44.5)	113 (41.1)		
Sex, n (%)					0.007	0.013
Female	556 (39.9)	224 (45.1)	223 (35.9)	109 (39.6)		
Male	838 (60.1)	273 (54.9)	399 (64.1)	166 (60.4)		
Marital status, n (%)					0.672	0.731
Unmarried	147 (10.5)	48 (9.7)	67 (10.8)	32 (11.6)		
Married	1,247 (89.5)	449 (90.3)	555 (89.2)	243 (88.4)		
Education level, n (%)					0.695	0.731
<High school	1,316 (94.4)	470 (94.6)	584 (93.9)	262 (95.3)		
≥High school	78 (5.6)	27 (5.4)	38 (6.1)	13 (4.7)		
Residence place, n (%)					<0.001	<0.001
Rural	968 (69.4)	378 (76.1)	415 (66.7)	175 (63.6)		
Urban	426 (30.6)	119 (23.9)	207 (33.3)	100 (36.4)		
Geographic region, n (%)					0.769	0.769
South	906 (65.0)	326 (65.6)	398 (64.0)	182 (66.2)		
North	488 (35.0)	171 (34.4)	224 (36.0)	93 (33.8)		
Smoking status, n (%)					0.039	0.061
Never	920 (66.0)	311 (62.6)	432 (69.5)	177 (64.4)		
Ever	100 (7.2)	31 (6.2)	47 (7.6)	22 (8.0)		
Now	374 (26.8)	155 (31.2)	143 (23.0)	76 (27.6)		
Drinking status, n (%)					0.001	0.002
Non-drinker	962 (69.0)	313 (63.0)	448 (72.0)	201 (73.1)		
Drinker	432 (31.0)	184 (37.0)	174 (28.0)	74 (26.9)		
Social activities, n (%)					0.106	0.148
No	1,173 (84.1)	432 (86.9)	513 (82.5)	228 (82.9)		
Yes	221 (15.9)	65 (13.1)	109 (17.5)	47 (17.1)		
Diabetes, n (%)					<0.001	<0.001
No	1,182 (84.8)	460 (92.6)	539 (86.7)	183 (66.5)		
Yes	212 (15.2)	37 (7.4)	83 (13.3)	92 (33.5)		
Cancer, n (%)					0.584	0.681
No	1,384 (99.3)	493 (99.2)	619 (99.5)	272 (98.9)		
Yes	10 (0.7)	4 (0.8)	3 (0.5)	3 (1.1)		
CLD, n (%)					0.083	0.122
No	1,229 (88.2)	448 (90.1)	548 (88.1)	233 (84.7)		
Yes	165 (11.8)	49 (9.9)	74 (11.9)	42 (15.3)		
Memory-related disease, n (%)					0.573	0.681
No	1,384 (99.3)	492 (99.0)	618 (99.4)	274 (99.6)		
Yes	10 (0.7)	5 (1.0)	4 (0.6)	1 (0.4)		
Digestive system disease, n (%)					0.008	0.014
No	928 (66.6)	305 (61.4)	436 (70.1)	187 (68.0)		
Yes	466 (33.4)	192 (38.6)	186 (29.9)	88 (32.0)		
CVD, n (%)					0.025	0.041
No	1,094 (78.5)	410 (82.5)	474 (76.2)	210 (76.4)		
Yes	300 (21.5)	87 (17.5)	148 (23.8)	65 (23.6)		

CTI, C-reactive protein–triglyceride–glucose index; CVD, cardiovascular disease; eGFR, estimated glomerular filtration rate; CLD, chronic lung disease; TC, total cholesterol; HDL-C, high-density lipoprotein cholesterol; LDL-C, low-density lipoprotein cholesterol; RC, remnant cholesterol; CRP, C-reactive protein; TG, triglyceride; FBG, fasting blood glucose.

^a^ *p*-Values for continuous variables calculated using ANOVA or Kruskal–Wallis test and for categorical variables using chi-squared test.

^b^ Adjusted *p*-values calculated using the Benjamini–Hochberg method.

### 3.3 Associations of CTI with CVD in the CHARLS and hospital-based cohorts

**Table 3** summarizes the associations of baseline CTI, cumCTI, and CTI change patterns with incident CVD in the CHARLS cohort. In the fully adjusted model, each 1-unit increase in baseline CTI was associated with a higher risk of incident CVD (HR = 1.251, 95% CI: 1.083–1.446, *p* = 0.002). Compared with that in Q1, the risk of incident CVD increased across baseline CTI quartiles, with the highest risk observed in Q4 (HR = 1.587, 95% CI: 1.257–2.003; *p* for trend < 0.001).

**Table 3:** Multivariable Cox regression of the relationships between CTI, cumCTI, different clusters of CTI, and the risk of new-onset CVD.

Characteristic	Model 1	*p*-value	Model 2	*p*-value	Model 3	*p*-value
	HR (95% CI)		HR (95% CI)		HR (95% CI)	
**CTI (per 1-unit increase)**	1.268 (1.126, 1.428)	**<0.001**	1.259 (1.117, 1.420)	**<0.001**	1.251 (1.083, 1.446)	**0.002**
Quartile CTI						
Q1	Ref.		Ref.		Ref.	
Q2	1.383 (1.117, 1.712)	**0.003**	1.333 (1.076, 1.651)	**0.008**	1.308 (1.054, 1.624)	**0.015**
Q3	1.580 (1.282, 1.948)	**<0.001**	1.526 (1.237, 1.882)	**<0.001**	1.468 (1.182, 1.823)	**<0.001**
Q4	1.650 (1.338, 2.034)	**<0.001**	1.612 (1.307, 1.988)	**<0.001**	1.587 (1.257, 2.003)	**<0.001**
*p* for trend		**<0.001**		**<0.001**		**<0.001**
**cumCTI (per 1-unit increase)**	1.108 (1.032, 1.190)	**0.005**	1.117 (1.040, 1.200)	**0.003**	1.118 (1.025, 1.220)	**0.012**
Quartile cumCTI						
Q1	Ref.		Ref.		Ref.	
Q2	1.084 (0.767, 1.532)	0.647	1.088 (0.770, 1.538)	0.632	1.082 (0.762, 1.535)	0.661
Q3	1.453 (1.046, 2.017)	**0.026**	1.435 (1.033, 1.996)	**0.032**	1.418 (1.009, 1.995)	**0.045**
Q4	1.503 (1.082, 2.089)	**0.015**	1.537 (1.106, 2.137)	**0.010**	1.504 (1.038, 2.179)	**0.031**
*p* for trend		**0.004**		**0.003**		**0.012**
CTI change						
Cluster 1	Ref.		Ref.		Ref.	
Cluster 2	1.401 (1.075, 1.826)	**0.013**	1.410 (1.081, 1.839)	**0.011**	1.402 (1.065, 1.844)	**0.016**
Cluster 3	1.469 (1.065, 2.026)	**0.019**	1.511 (1.095, 2.085)	**0.012**	1.443 (1.001, 2.081)	**0.049**
*p* for trend		**0.010**		**0.006**		**0.026**

Model 1, unadjusted for any covariates; Model 2, adjusted for age, sex, and marital status; Model 3, adjusted for age, sex, marital status, education level, residence place, geographic region, smoking status, drinking status, sleep duration, social activities, diabetes, cancer, chronic lung disease, memory-related disease, digestive system disease, eGFR, and RC.

CTI, C-reactive protein–triglyceride–glucose index; CVD, cardiovascular disease; HR, hazard ratio; CI, confidence interval; Q, quartile; Ref., reference; eGFR, estimated glomerular filtration rate; RC, remnant cholesterol.

Bold values indicate P < 0.05.

The cumCTI and CTI change-pattern analyses used Wave 3 in 2015 as the time origin and evaluated incident CVD events occurring after the repeated CTI measurements had been completed. In exploratory repeated-measures analyses, cumCTI was positively associated with incident CVD in the CHARLS cohort. For each 1-unit increase in cumCTI, the hazard of incident CVD increased significantly (HR = 1.118, 95% CI: 1.025–1.220, *p* = 0.012). Compared with participants in Q1, those in Q4 had a higher hazard of incident CVD (HR = 1.504, 95% CI: 1.038–2.179, *p* = 0.031), and a significant dose–response trend was observed across quartiles (*p* for trend = 0.012).

For CTI change patterns, both Cluster 2 and Cluster 3 were associated with higher CVD risk than Cluster 1 (Cluster 2: HR = 1.402, 95% CI: 1.065–1.844, *p* = 0.016; Cluster 3: HR = 1.443, 95% CI: 1.001–2.081, *p* = 0.049; *p* for trend = 0.026).

In the hospital-based cross-sectional replication cohort, baseline CTI was associated with prevalent CVD (**Table 4**). Each 1-unit increase in baseline CTI was associated with higher odds of prevalent CVD (OR = 1.798, 95% CI: 1.627–1.988, *p* < 0.001), and the highest quartile showed the strongest association compared with Q1 (Q4: OR = 2.193, 95% CI: 1.898–2.537; *p* for trend < 0.001).

**Table 4:** Multivariable logistic regression of the association between baseline CTI and prevalent CVD in the hospital-based cross-sectional replication cohort.

Characteristic	Model 1	*p*-Value	Model 2	*p*-value	Model 3	*p*-value
	OR (95% CI)		OR (95% CI)		OR (95% CI)	
CVD by CTI						
**CTI (per 1-unit increase)**	2.202 (2.012, 2.413)	**<0.001**	1.976 (1.800, 2.171)	**<0.001**	1.798 (1.627, 1.988)	**<0.001**
Quartile CTI						
Q1	Ref.		Ref.		Ref.	
Q2	1.536 (1.339, 1.762)	**<0.001**	1.437 (1.251, 1.652)	**<0.001**	1.376 (1.195, 1.585)	**<0.001**
Q3	1.979 (1.729, 2.265)	**<0.001**	1.803 (1.572, 2.069)	**<0.001**	1.657 (1.440, 1.908)	**<0.001**
Q4	2.908 (2.545, 3.326)	**<0.001**	2.504 (2.183, 2.874)	**<0.001**	2.193 (1.898, 2.537)	**<0.001**
***p* for trend**		**<0.001**		**<0.001**		**<0.001**

Model 1, unadjusted for any covariates; Model 2, adjusted for age, sex, and marital status; Model 3, adjusted for age, sex, marital status, education level, smoking status, drinking status, diabetes, cancer, chronic lung disease, memory-related disease, digestive system disease, eGFR, and RC.

CTI, C-reactive protein–triglyceride–glucose index; CVD, cardiovascular disease; OR, odds ratio; CI, confidence interval; Q, quartile; Ref., reference; eGFR, estimated glomerular filtration rate; RC, remnant cholesterol.

Bold values indicate P < 0.05.

### 3.4 Kaplan–Meier survival curve analysis

**Figure 3:**
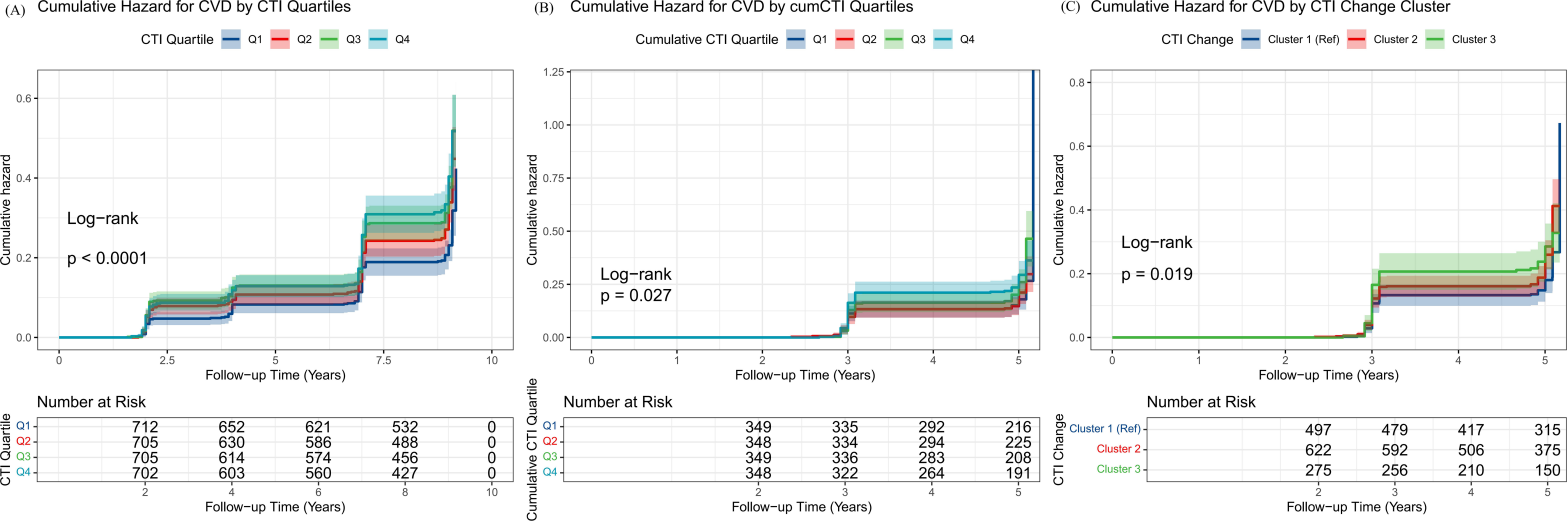
Cumulative hazard curves for incident CVD according to baseline CTI quartiles **(A)**, cumCTI quartiles **(B)**, and CTI level-change clusters **(C)**. CTI, C-reactive protein–triglyceride–glucose index; CVD, cardiovascular disease.

**Figure 3** presents cumulative hazard curves for incident CVD according to baseline CTI quartiles, cumCTI quartiles, and CTI level-change clusters. Significant between-group differences were observed for baseline CTI quartiles (log-rank *p* < 0.0001), cumCTI quartiles (log-rank *p* = 0.027), and CTI level-change clusters (log-rank *p* = 0.019).

### 3.5 RCS analysis

**Figure 4:**
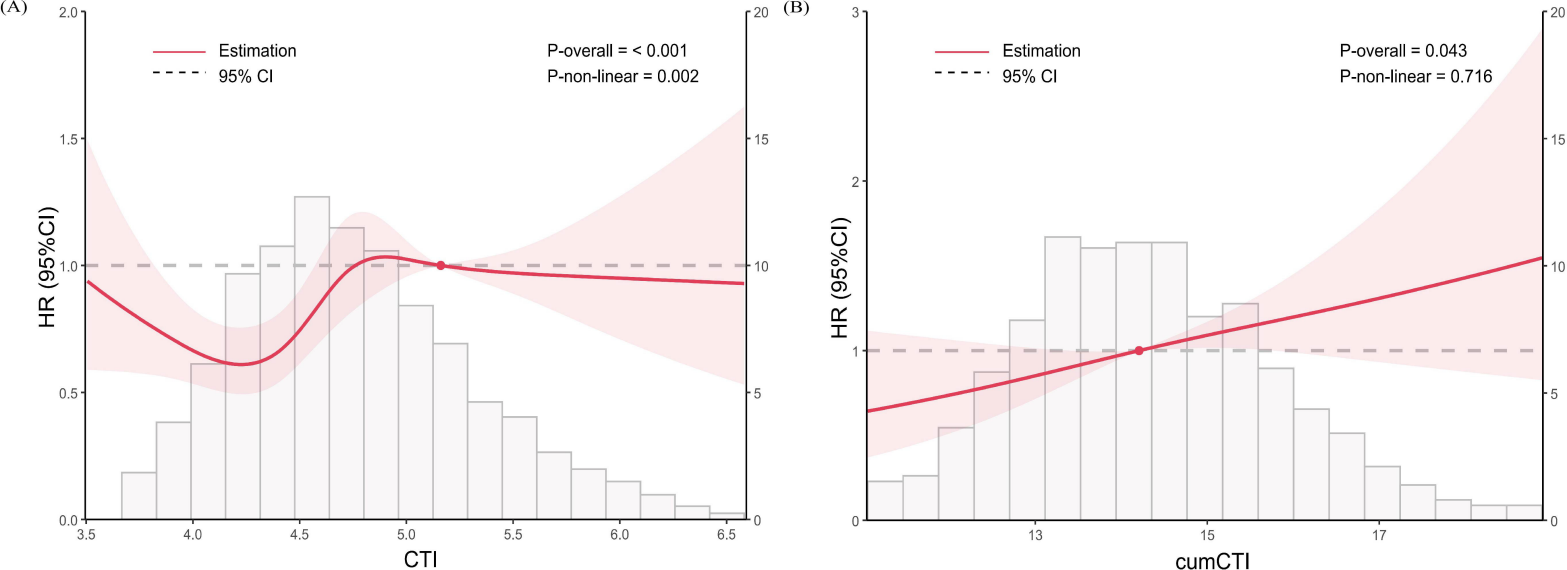
Dose–response associations between **(A)** baseline CTI and **(B)** cumCTI and incident CVD. Restricted cubic spline Cox models with three knots at the 10th, 50th, and 90th percentiles of each exposure, adjusted for age, sex, marital status, education level, residence place, geographic region, smoking status, drinking status, sleep duration, social activities, diabetes, cancer, chronic lung disease, memory-related disease, digestive system disease, eGFR, and RC. Solid lines depict hazard ratios (HRs); shaded bands show 95% confidence intervals (CIs). CTI, C-reactive protein–triglyceride–glucose index; HR, hazard ratio; CI, confidence interval; eGFR, estimated glomerular filtration rate; RC, remnant cholesterol; CVD, cardiovascular disease.

RCS analysis showed a non-linear association between baseline CTI and incident CVD in the CHARLS cohort (overall *p* < 0.001; *p* for non-linearity = 0.002; **Figure 4A**). Threshold-effect analysis identified an inflection point at CTI = 4.897 (**Supplementary Table 7**). Below this threshold, baseline CTI was positively associated with incident CVD (HR = 1.577, 95% CI: 1.218–2.042, *p* < 0.001), whereas the association was not significant above the threshold (HR = 0.984, 95% CI: 0.754–1.284, *p* = 0.902). In contrast, cumCTI showed an approximately linear positive association with incident CVD (overall *p* = 0.043; *p* for non-linearity = 0.716; **Figure 4B**). In the hospital-based cohort, baseline CTI showed a positive dose–response association with prevalent CVD (**Supplementary Figure 2**).

### 3.6 Subgroup analysis

**Figure 5:**
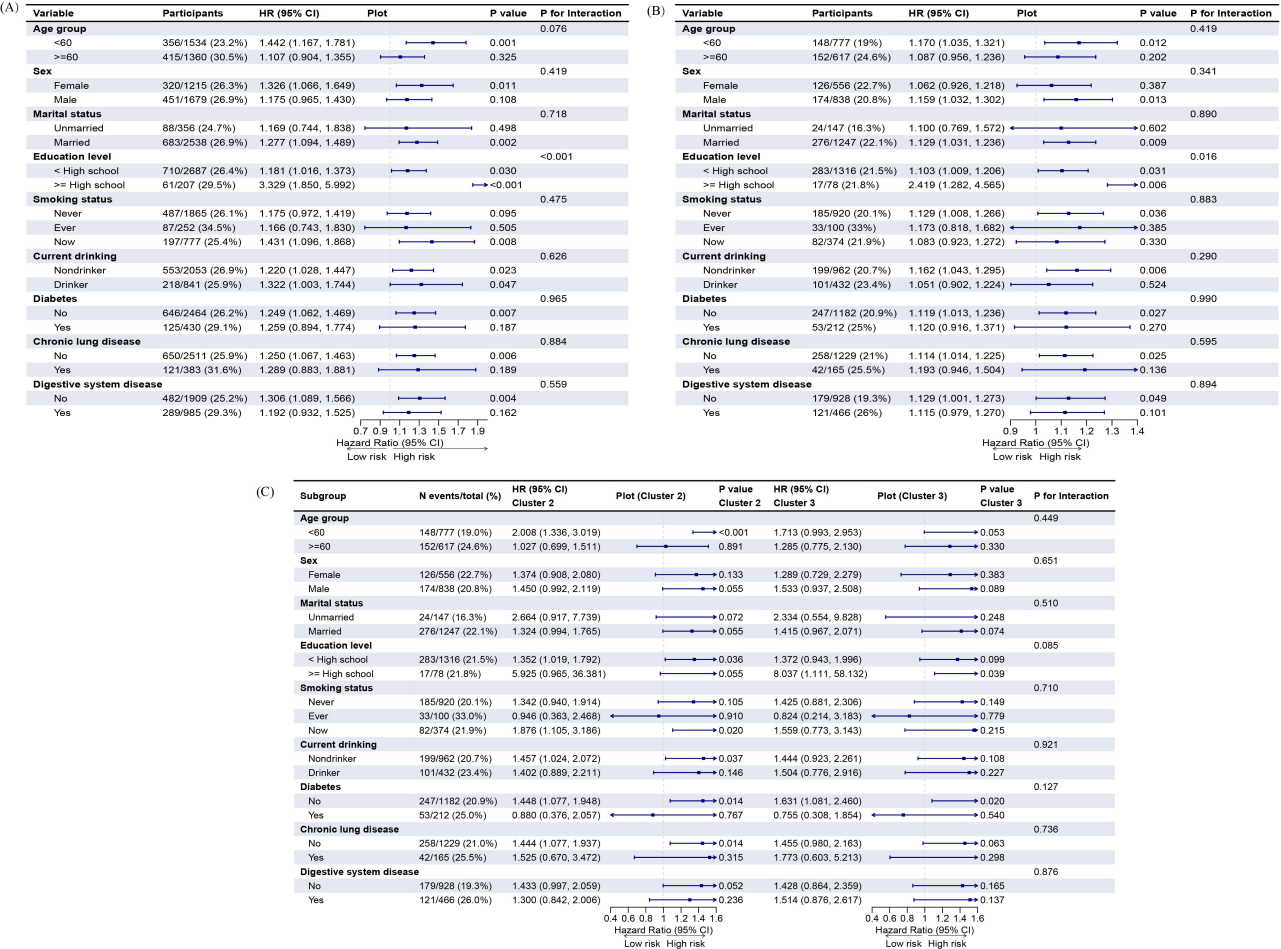
Subgroup and interaction analyses of the associations of baseline CTI, cumCTI, and CTI level-change patterns with incident CVD. **(A)** Baseline CTI, **(B)** cumCTI, and **(C)** CTI change patterns (Cluster 1 as reference). Hazard ratios and 95% CIs are from fully adjusted Cox models (Model 3). Adjusted for age, sex, marital status, education level, residence place, geographic region, smoking status, drinking status, sleep duration, social activities, diabetes, cancer, chronic lung disease, memory-related disease, digestive system disease, eGFR, and RC. CTI, C-reactive protein–triglyceride–glucose index; HR, hazard ratio; CI, confidence interval; eGFR, estimated glomerular filtration rate; RC, remnant cholesterol; CVD, cardiovascular disease.

Subgroup analyses showed that the associations of baseline CTI and cumCTI with incident CVD were directionally consistent across most predefined strata (**Figure 5**). For both baseline CTI and cumCTI, a significant interaction was observed only for education level (*p* for interaction < 0.001 and *p* for interaction = 0.016, respectively). For CTI level-change patterns, no statistically significant interaction was detected across the predefined subgroups.

### 3.7 Comparative discrimination, decision curve, and incremental performance analyses

Exploratory comparative performance analyses are shown in **Supplementary Figure 3**; **Supplementary Table 8**. In the time-dependent ROC analysis for 9-year incident CVD, CTI showed an area under the curve (AUC) of 0.556 (95% CI: 0.529–0.583), compared with 0.554 (95% CI: 0.528–0.580) for CRP, 0.523 (95% CI: 0.496–0.547) for TG, and 0.508 (95% CI: 0.477–0.534) for FBG (**Supplementary Figure 3A**). DCA showed the net benefit curves for CTI, CRP, FBG, and TG across threshold probabilities (**Supplementary Figure 3B**). Adding CTI to the basic model changed Harrell’s C-index from 0.615 (95% CI: 0.594–0.636) to 0.618 (95% CI: 0.597–0.638; *p* = 0.378), with a continuous NRI of 0.157 (95% CI: 0.059–0.257; *p* = 0.002) and an IDI of 0.003 (95% CI: 0.001–0.005; *p* = 0.006) (**Supplementary Table 8**).

### 3.8 Sensitivity analyses

Sensitivity analyses are summarized in **Supplementary Tables 9**–**S13**. The association between baseline CTI and incident CVD remained statistically significant after excluding early CVD cases, restricting the analysis to complete covariate data, and excluding deaths during follow-up. In models additionally adjusted for BMI and PA, the continuous CTI association was attenuated, whereas the highest CTI quartile remained associated with incident CVD. For cumCTI and CTI level-change patterns, associations were directionally consistent in several sensitivity analyses but were attenuated after complete-case restriction and additional adjustment for BMI and PA. In the joint model including both baseline CTI and delta CTI, baseline CTI remained associated with incident CVD, whereas delta CTI was not independently associated.

## 4 Discussion

In this study, higher CTI-related inflammatory-metabolic burden was associated with greater CVD risk among middle-aged and older adults with arthritis in a nationwide prospective cohort. In the CHARLS, baseline CTI was consistently associated with incident CVD, whereas cumCTI and exploratory two-time-point CTI level-change patterns provided supportive but more exploratory evidence. RCS analyses showed a non-linear association for baseline CTI and an approximately linear association for cumCTI. The hospital-based cohort further supported the cross-sectional association between baseline CTI and prevalent CVD; however, this component should be interpreted as replication of association rather than validation of prognostic performance.

Our findings are consistent with increasing evidence that metabolic-inflammatory indices are associated with CVD risk. The TyG index has been linked to new-onset CVD and mortality across different populations (28, 29). Studies integrating TyG with inflammatory markers have further suggested potential value for identifying individuals at higher cardiovascular risk (15, 30). Biologically, CTI may reflect the combined contribution of systemic inflammation and IR to vascular injury and atherosclerotic progression. In arthritis, persistent inflammatory activation may promote endothelial dysfunction and vascular injury (31, 32). Proinflammatory cytokine activity, including IL-6 and TNF-α, may further contribute to systemic inflammation beyond joint damage (33). Meanwhile, the TyG component of CTI is a surrogate marker of IR, which has been associated with adverse cardiovascular outcomes (34–36). By integrating CRP and TyG, CTI may capture overlapping inflammatory and metabolic burden more comprehensively than either component alone (37). Previous studies have also linked higher CTI values to CHD, stroke, and mortality among individuals with CVD (14, 38). Our study extends this evidence to adults with arthritis, a population in whom inflammatory burden and metabolic comorbidities may jointly contribute to cardiovascular vulnerability.

The cumCTI analysis suggested that cumulative inflammatory-metabolic burden across two biomarker waves may be related to subsequent CVD risk, consistent with evidence from other cumulative or composite metabolic indices (39, 40). Nevertheless, cumCTI was derived from only two CTI measurements, and some estimates were attenuated in sensitivity analyses. Therefore, this finding should be interpreted as exploratory rather than definitive evidence that cumulative CTI provides additional information beyond a single baseline CTI measurement. Future studies with more repeated biomarker measurements are needed to clarify whether long-term CTI exposure offers incremental information over a single baseline measurement.

Subgroup analyses suggested that the CTI–CVD association was generally directionally consistent across most predefined strata, with education level being the only subgroup variable showing a statistically significant interaction for both baseline CTI and cumCTI. Although age, socioeconomic status, and smoking may influence inflammatory-metabolic cardiovascular risk through competing risks, healthcare engagement, psychosocial stress, and smoking-related metabolic injury pathways, these factors did not consistently modify the CTI–CVD association in our analysis (41–43). Given the exploratory nature of subgroup analyses and the issue of multiple comparisons, these findings should be interpreted cautiously and require confirmation in future studies.

The exploratory two-time-point CTI level-change patterns provided an additional but limited perspective on cardiovascular risk assessment. Because the clustering procedure was based on only two CTI measurements, these patterns should not be interpreted as complex longitudinal trajectories. Rather, they likely reflect a combination of overall CTI magnitude and limited short-term change, with differences in CTI burden contributing substantially to cluster separation. This interpretation is supported by the joint model including baseline CTI and delta CTI, in which baseline CTI remained associated with incident CVD, whereas delta CTI was not independently associated. Although previous evidence suggests that sustained IR-related burden may contribute to cardiovascular risk (44), our findings do not establish short-term CTI change itself as an independent predictor beyond baseline CTI.

In the RCS analysis, baseline CTI showed a non-linear association with incident CVD, with an estimated inflection point at approximately 4.897. This finding suggests that cardiovascular risk may increase more rapidly when CTI rises from a relatively low to an intermediate range, whereas the marginal increase in risk appears to attenuate at higher CTI levels. Similar non-linear patterns have been reported for inflammatory and metabolic markers in relation to cardiovascular outcomes (45). However, this inflection point should not be regarded as a definitive clinical cutoff or intervention threshold because it was derived from an observational cohort and requires validation in independent prospective populations (46). Thus, CTI may be considered as part of an early risk-assessment framework rather than as a standalone decision-making threshold.

Previous studies have shown that metabolic and inflammatory biomarkers influence cardiovascular outcomes (47). CTI has also been proposed as a composite marker linking metabolic and inflammatory processes (14, 48). By evaluating baseline CTI together with cumCTI and exploratory two-time-point CTI level-change patterns, our study provides additional evidence that inflammatory-metabolic burden may be relevant to cardiovascular risk in adults with arthritis. Nevertheless, the repeated-measures findings remain exploratory, and the modest incremental predictive performance observed in this study suggests that CTI should currently be viewed as a complementary marker rather than a standalone clinical prediction tool.

## 5 Strengths and limitations

This study has several strengths. First, it used a nationwide prospective cohort of middle-aged and older adults, allowing us to examine the association between CTI and incident CVD over follow-up. Second, baseline CTI, cumCTI, and exploratory two-time-point CTI level-change patterns were assessed together, providing a broader description of inflammatory-metabolic burden than single-time-point analysis alone. Third, a hospital-based cohort was included to examine whether the association between baseline CTI and prevalent CVD could also be observed in an independent clinical setting. RCS, subgroup, sensitivity, included/excluded participant comparison, and exploratory predictive performance analyses were also performed. Our findings may have clinical relevance because CTI is derived from inexpensive and routinely available biomarkers. However, the exploratory predictive performance analyses showed only modest improvement. Although CTI slightly increased the C-index and showed modest improvement in NRI and IDI, ROC analysis and DCA suggested limited incremental value. Therefore, CTI should currently be viewed as a complementary marker for recognizing inflammatory-metabolic cardiovascular vulnerability rather than a standalone clinical prediction tool.

Several limitations should be acknowledged. First, CVD outcomes in the CHARLS were based on self-reported physician-diagnosed heart disease and/or stroke rather than centrally adjudicated endpoints. This may have introduced outcome misclassification and limited our ability to distinguish specific cardiovascular event subtypes. Although such misclassification was likely to be predominantly non-differential with respect to CTI levels, differential misclassification cannot be fully excluded. In contrast, prevalent CVD in the hospital-based cross-sectional replication cohort was identified from hospital medical records rather than self-report, which reduced reliance on participant recall. However, because the hospital-based cohort was cross-sectional and CVD events were not centrally adjudicated by a standardized endpoint committee, diagnostic heterogeneity may still exist. Future prospective studies should validate CVD outcomes using adjudicated endpoints, medication information, cardiovascular imaging, and standardized clinical records. Second, arthritis status in the CHARLS was based on self-reported physician diagnosis of “arthritis or rheumatism”, rather than rheumatologist-confirmed clinical classification. This definition may include heterogeneous conditions, such as OA, RA, gout, spondyloarthritis, psoriatic arthritis, and non-specific rheumatic or musculoskeletal disorders. Therefore, the present findings should not be interpreted as specific to immune-mediated inflammatory arthritis or autoimmune arthritis. Third, cumCTI and CTI level-change patterns were derived from only two CTI measurements. Thus, the K-means clusters should be interpreted as exploratory two-time-point CTI level-change patterns rather than true longitudinal trajectories. The independent contribution of CTI change beyond baseline CTI could not be fully determined, and future studies with more repeated biomarker measurements are needed. Fourth, residual confounding cannot be fully excluded. Although multiple demographic, lifestyle-related, metabolic, and comorbidity variables were adjusted for, unmeasured factors such as diet, medication use, arthritis disease activity, genetic susceptibility, and other clinical characteristics may have influenced the results. PA was considered only in the sensitivity analysis because of substantial structural missingness. Fifth, the hospital-based cohort was cross-sectional and used prevalent rather than incident CVD as the outcome. Therefore, this component should be interpreted as cross-sectional replication of the association between baseline CTI and prevalent CVD, rather than validation of CTI for predicting future cardiovascular events. Finally, selection bias may have occurred because participants with missing key information were excluded, although included and excluded participants were compared. The findings may be most applicable to middle-aged and older Chinese adults with arthritis, and further validation in other populations is warranted.

## 6 Conclusion

In middle-aged and older adults with arthritis, higher CTI-related inflammatory-metabolic burden was associated with increased CVD risk. The hospital-based cohort further supported the association between baseline CTI and prevalent CVD. As a low-cost and readily accessible composite biomarker, CTI may provide useful information for recognizing cardiovascular vulnerability in this heterogeneous arthritis population. However, its incremental predictive value appeared modest, and the repeated-measures findings should be interpreted as exploratory. Further prospective studies with adjudicated CVD outcomes, repeated biomarker measurements, detailed arthritis phenotyping, and formal predictive performance analyses are warranted to clarify its clinical utility.

## Data Availability

The raw data supporting the conclusions of this article will be made available by the authors, without undue reservation.
