# Supplementary material for: Association between the C-reactive protein–triglyceride–glucose index and incident cardiovascular disease in middle-aged and older adults with arthritis: a nationwide prospective cohort study with hospital-based cross-sectional replication
Source: Front Immunol. 2026 Jun 10;17:1854928. doi: 10.3389/fimmu.2026.1854928 (PMC13290972; doi:10.3389/fimmu.2026.1854928)
Supplement: Supplementary file 1 [file SupplementaryFile1.docx]

**Supplementary Material**

**Text S1.** R code for Computing Time-Weighted Cumulative CTI (cumCTI).

**Text S2.** Definition of variables involved in this study.

**Figure S1.** Flow chart of participant selection in the hospital-based cross-sectional replication cohort.

**Figure S2.** Dose-response association between baseline CTI and prevalent CVD in the hospital-based cross-sectional replication cohort.

**Figure S3.** Comparative discrimination and decision curve analysis for incident CVD.

**Table S1.** Proportion of missing covariate data.

**Table S2.** Characteristics of included and excluded participants in the baseline CTI cohort.

**Table S3.** Characteristics of included and excluded participants in the cumCTI and CTI level-change pattern cohort.

**Table S4.** Characteristics of included and excluded participants in the hospital-based cross-sectional replication cohort.

**Table S5.** Variance inflation factors (VIFs) for covariates in fully adjusted Cox models (Model 3).

**Table S6.** Baseline characteristics according to baseline CTI quartiles in the hospital-based cross-sectional replication cohort.

**Table S7.** Threshold effect analysis of baseline CTI with new-onset CVD.

**Table S8.** Incremental predictive performance for 9-year CVD in the CHARLS arthritis cohort.

**Table S9.** Sensitivity analysis excluding participants who developed CVD within the first 2 years of follow-up.

**Table S10.** Sensitivity analysis including only participants with complete covariate data.

**Table S11.** Sensitivity analysis excluding participants who died during follow-up.

**Table S12.** Sensitivity analysis further adjusted for BMI and physical activity.

**Table S13.** Sensitivity analysis jointly adjusted for baseline CTI and delta CTI.

**Text S1.** R code for Computing Time-Weighted Cumulative CTI (cumCTI)

#------ 1. Purpose & Environment -------

# Purpose: Calculate time-weighted cumulative exposure (cumCTI) from two CTI

# measurements (CTI_w1 = 2012, CTI_w3 = 2015) in data-frame dt.

# Formula: cumCTI = (CTI_2012 + CTI_2015) / 2 * (2015 - 2012)

# Depends: Base R only; dplyr optional for additional cleaning.

#------ 2. Data Import & Cleaning -------

# Assume dt exists and contains numeric or coercible CTI_w1 and CTI_w3.

stopifnot(exists("dt"))

stopifnot(all(c("CTI_w1", "CTI_w3") %in% names(dt)))

# Safe numeric conversion

dt$CTI_w1 <- suppressWarnings(as.numeric(dt$CTI_w1))

dt$CTI_w3 <- suppressWarnings(as.numeric(dt$CTI_w3))

#------ 3. Analysis / Modelling --------

delta_years <- 2015 - 2012 # 3-year interval

# Trapezoidal approximation; NA if either CTI missing

dt$cumCTI <- ((dt$CTI_w1 + dt$CTI_w3) / 2) * delta_years

# Optional inspection: summary(dt$cumCTI)

#------ 4. Output ----------------------

# For reproducibility no file is written.

# Example export (UTF-8, blank for NA):

# write.csv(dt, file = "cumCTI_output.csv", row.names = FALSE, fileEncoding = "UTF-8", na = "")

**Text S2.** Definition of variables involved in this study.

The residence place was categorized as rural or urban. Diabetes was defined as FBG ≥ 7.0 mmol/L, HbA1c ≥ 6.5% or self-reported diabetes diagnosis. The estimated glomerular filtration rate (eGFR) was calculated based on the result of a multicenter study in Chinese populations ^[1]^.

The equation is eGFR = 173.9×CysC− 0.725×Cr− 0.184×Age− 0.193 × 0.89 [if female]. The coefficients of variation were less than 4.0% for triglycerides and high-density lipoprotein cholesterol (HDL-C), under 3.0% for total cholesterol, and below 5.0% for fasting glucose. Non-HDL-C was calculated by subtracting HDL-C from total cholesterol, while remnant cholesterol (RC) was defined as non-HDL-C minus low-density lipoprotein cholesterol (LDL-C) ^[2]^.

**References**

[1] Huang YC, Chen SL, Dong Y, Shi Y. Association between elevated serum uric acid levels and high estimated glomerular filtration rate with reduced risk of low muscle strength in older people: a retrospective cohort study. BMC Geriatr. 2023;23(1):652.

[2] Wang Y, Bi L, Li Q, Wang Q, Lv T, Zhang P. Remnant cholesterol inflammatory index and its association with all-cause and cause-specific mortality in middle-aged and elderly populations: evidence from US and Chinese national population surveys. *Lipids Health Dis*. 2025;24(1):155.

**Figure S1.** Flow chart of participant selection in the hospital-based cross-sectional replication cohort.


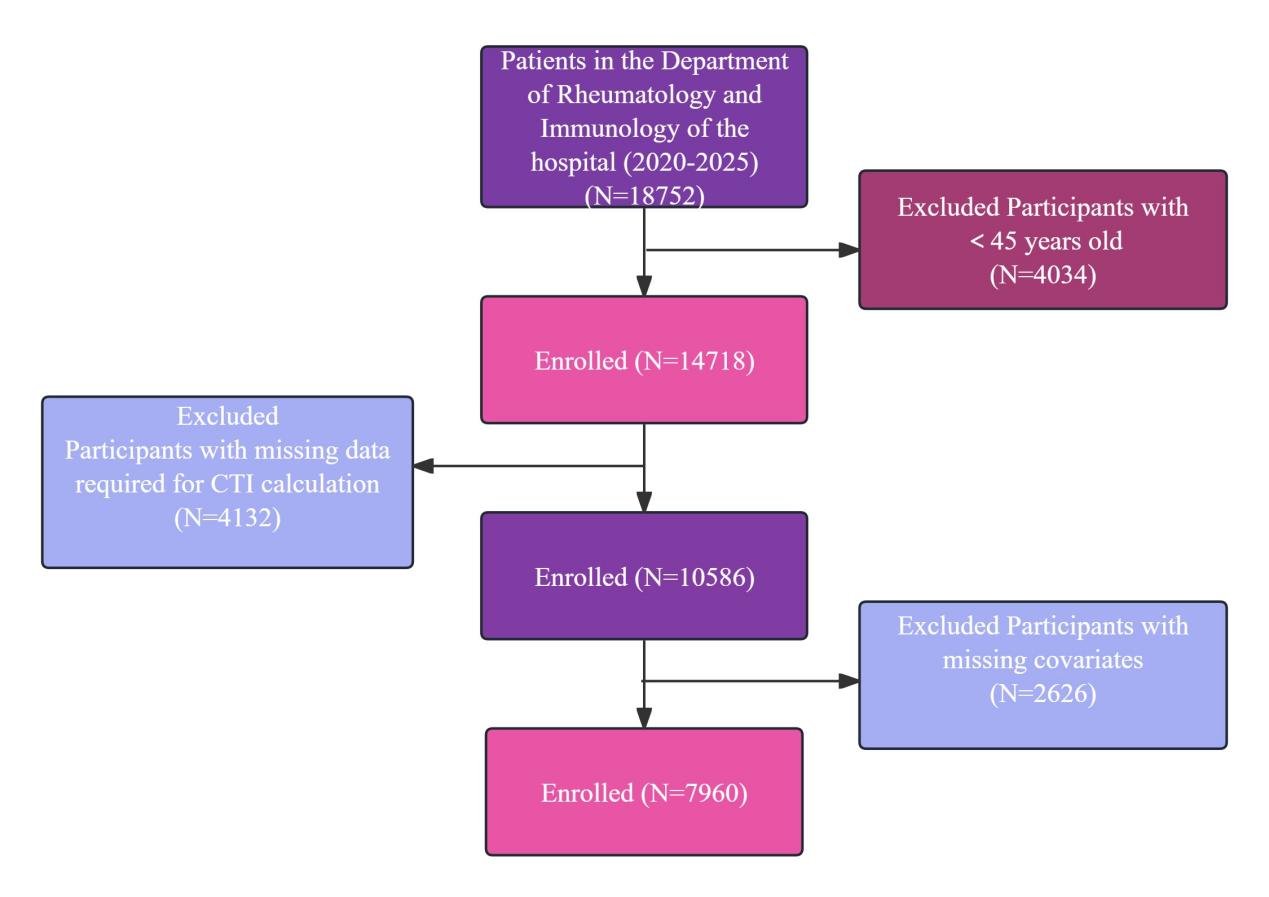


**Figure S2.** Dose-response association between baseline CTI and prevalent CVD in the hospital-based cross-sectional replication cohort.


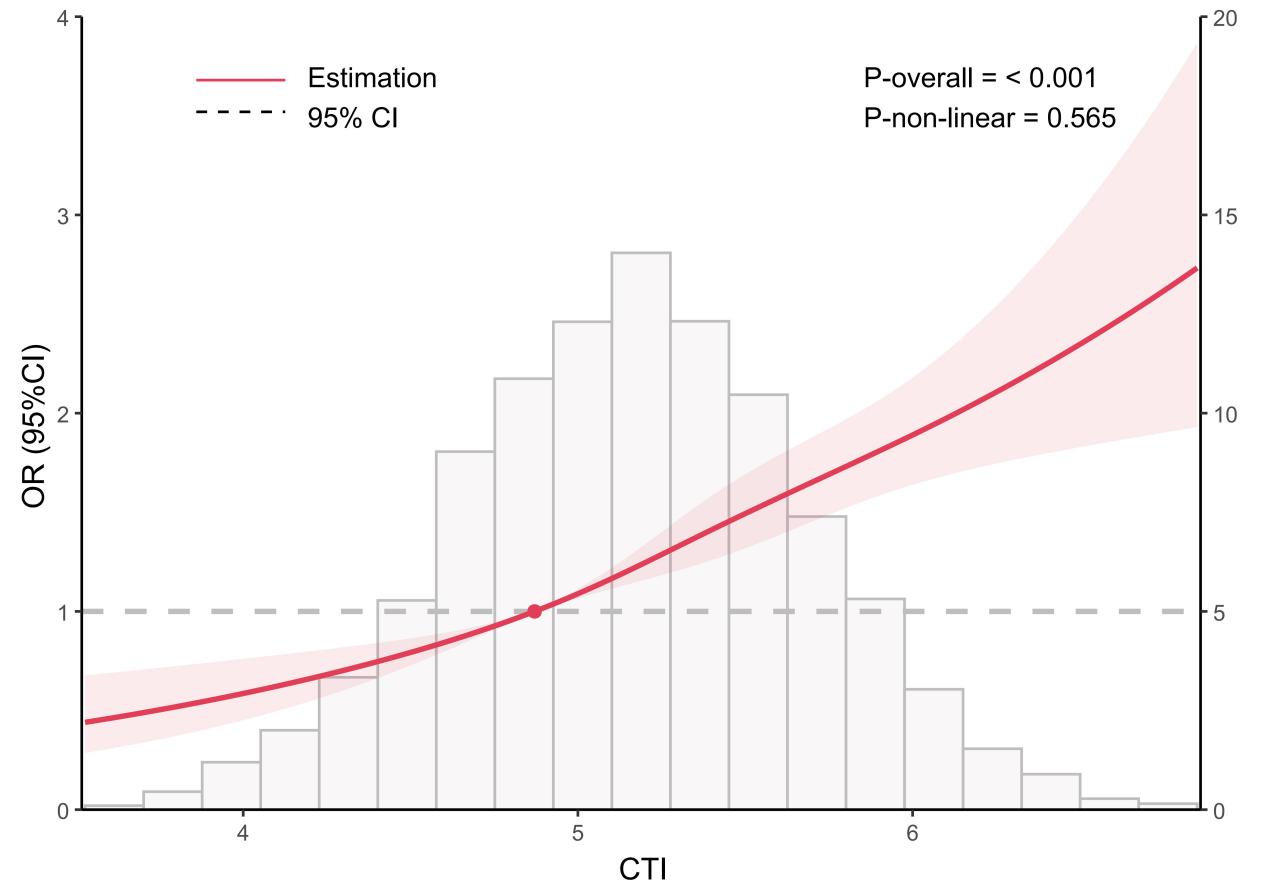


**Figure S3.** Comparative discrimination and decision curve analysis for incident CVD.


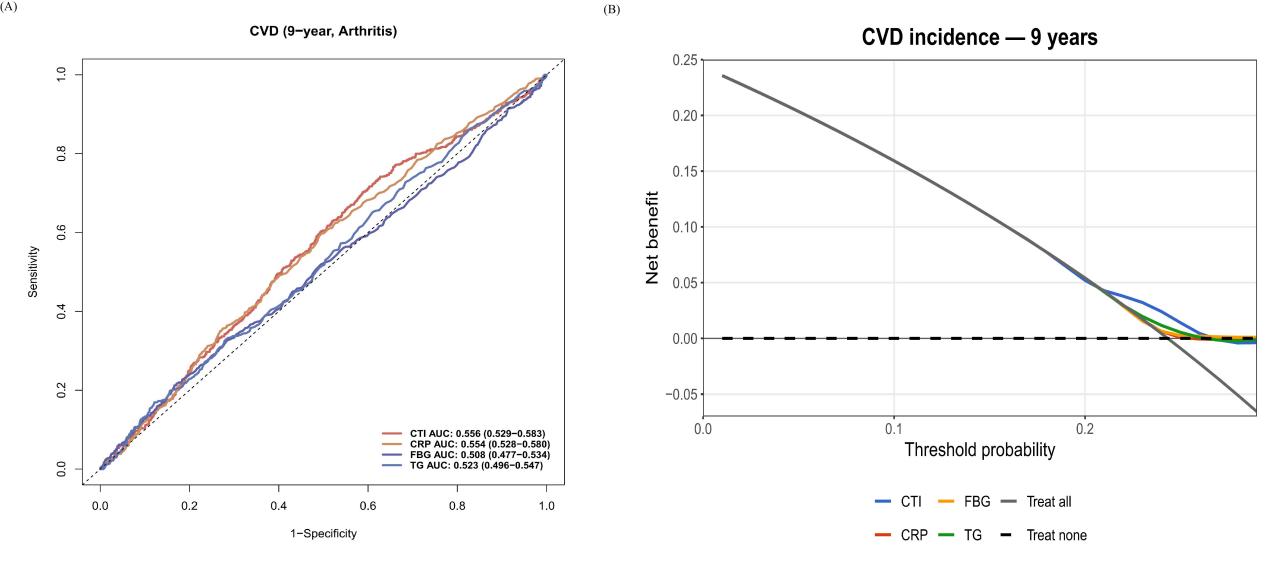


**Table S1.** Proportion of missing covariate data.

| **Characteristic** | **Missing proportion (N=1394)** | **Missing proportion (N=2894)** |
| --- | --- | --- |
| **Age** | 0% | 0% |
| **Sleep duration** | 3.3% | 4.35% |
| **eGFR** | 0.29% | 0.21% |
| **TC** | 0% | 0% |
| **HDL-C** | 0% | 0% |
| **LDL-C** | 0.29% | 0.24% |
| **RC** | 0.29% | 0.24% |
| **Sex** | 0.22% | 0.14% |
| **Marital status** | 0% | 0% |
| **Education level** | 0.14% | 0.1% |
| **Geographic region** | 0% | 0% |
| **Smoking status** | 0% | 0.03% |
| **Drinking status** | 6.17% | 6.91% |
| **Diabetes** | 0% | 0% |
| **Cancer** | 0.93% | 0.79% |
| **CLD** | 0.22% | 0.41% |
| **Liver disease** | 0.86% | 0.9% |
| **Stomach disease** | 0.22% | 0.41% |

Abbreviations: eGFR, estimated glomerular filtration rate; CLD, chronic lung disease; TC, total cholesterol; HDL-C, high-density lipoprotein cholesterol; LDL-C, low-density lipoprotein cholesterol; RC, remnant cholesterol; Digestive system disease in the main analyses was defined based on liver disease and stomach disease.

**Table S2.** Characteristics of included and excluded participants in the baseline CTI cohort.

| **Variable** | **Included (n=2894)** | **Excluded (n=14814)** | ***P*-value** |
| --- | --- | --- | --- |
| **Age, years** | 59.62 ± 9.09 | 58.87 ± 10.35 | <0.001 |
| **Sleep duration, h** | 6.11 ± 2.01 | 6.43 ± 1.87 | <0.001 |
| **eGFR, ml/min/1.73 m²** | 95.67 ± 14.32 | 95.71 ± 14.75 | 0.901 |
| **TC, mg/dL** | 195.82 ± 39.19 | 192.02 ± 38.75 | <0.001 |
| **HDL-C, mg/dL** | 52.49 ± 15.39 | 50.30 ± 15.27 | <0.001 |
| **LDL-C, mg/dL** | 117.56 ± 34.62 | 115.48 ± 34.99 | 0.005 |
| **RC, mg/dL** | 25.26 ± 24.04 | 26.04 ± 24.82 | 0.135 |
| **Baseline CRP, mg/L** | 2.72 ± 6.80 | 2.83 ± 7.89 | 0.464 |
| **Baseline TG, mg/dL** | 131.51 ± 114.09 | 136.03 ± 108.95 | 0.062 |
| **Baseline FBG, mg/dL** | 108.74 ± 31.79 | 110.29 ± 36.34 | 0.032 |
| **Baseline CTI** | 4.75 ± 0.57 | 4.77 ± 0.60 | 0.300 |
| **Age group, n (%)** |  |  | <0.001 |
| <60 | 1534 (53.0%) | 8482 (57.4%) |  |
| >=60 | 1360 (47.0%) | 6306 (42.6%) |  |
| **Sex, n (%)** |  |  | <0.001 |
| Female | 1215 (42.0%) | 7256 (49.0%) |  |
| Male | 1675 (58.0%) | 7546 (51.0%) |  |
| **Marital status, n (%)** |  |  | 0.421 |
| Unmarried | 356 (12.3%) | 1902 (12.9%) |  |
| Married | 2538 (87.7%) | 12879 (87.1%) |  |
| **Education level, n (%)** |  |  | <0.001 |
| < High school | 2684 (92.8%) | 12723 (86.2%) |  |
| >= High school | 207 (7.2%) | 2039 (13.8%) |  |
| **Residence place, n (%)** |  |  | <0.001 |
| Rural | 1988 (68.7%) | 8549 (57.7%) |  |
| Urban | 906 (31.3%) | 6262 (42.3%) |  |
| **Geographic region, n (%)** |  |  | <0.001 |
| South | 1918 (66.3%) | 7948 (53.7%) |  |
| North | 976 (33.7%) | 6863 (46.3%) |  |
| **Smoking status, n (%)** |  |  | <0.001 |
| Never | 1864 (64.4%) | 8762 (59.8%) |  |
| Ever | 252 (8.7%) | 1817 (12.4%) |  |
| Now | 777 (26.9%) | 4085 (27.9%) |  |
| **Drinking status, n (%)** |  |  | 0.894 |
| Nondrinker | 1940 (72.0%) | 9857 (72.2%) |  |
| Drinker | 754 (28.0%) | 3803 (27.8%) |  |
| **Social activities, n (%)** |  |  | 0.105 |
| No | 2438 (84.2%) | 12294 (83.0%) |  |
| Yes | 456 (15.8%) | 2520 (17.0%) |  |
| **Diabetes, n (%)** |  |  | 0.063 |
| No | 2705 (95.0%) | 13707 (94.1%) |  |
| Yes | 141 (5.0%) | 853 (5.9%) |  |
| **Cancer, n (%)** |  |  | 0.130 |
| No | 2853 (99.4%) | 14476 (99.1%) |  |
| Yes | 18 (0.6%) | 137 (0.9%) |  |
| **CLD, n (%)** |  |  | <0.001 |
| No | 2499 (86.7%) | 13181 (90.2%) |  |
| Yes | 383 (13.3%) | 1431 (9.8%) |  |
| **Memory-related disease, n (%)** |  |  | 0.205 |
| No | 2843 (98.6%) | 14374 (98.3%) |  |
| Yes | 39 (1.4%) | 249 (1.7%) |  |
| **Digestive system disease, n (%)** |  |  | <0.001 |
| No | 1959 (68.0%) | 11531 (78.8%) |  |
| Yes | 923 (32.0%) | 3102 (21.2%) |  |

Abbreviations: CTI, C-reactive protein-triglyceride-glucose index; eGFR, estimated glomerular filtration rate; CLD, chronic lung disease; TC, total cholesterol; HDL-C, high-density lipoprotein cholesterol; LDL-C, low-density lipoprotein cholesterol; RC, remnant cholesterol; CRP, C-reactive protein; TG, triglyceride; FBG, fasting blood glucose. Digestive system disease in the main analyses was defined based on liver disease and stomach disease.

**Table S3.** Characteristics of included and excluded participants in the cumCTI and CTI level-change pattern cohort.

| **Variable** | **Included (n=1394)** | **Excluded (n=16314)** | ***P*-value** |
| --- | --- | --- | --- |
| **Age, years** | 58.65 ± 8.19 | 59.02 ± 10.31 | 0.119 |
| **Sleep duration, h** | 6.09 ± 1.99 | 6.40 ± 1.89 | <0.001 |
| **eGFR, ml/min/1.73 m²** | 97.20 ± 13.01 | 95.49 ± 14.84 | <0.001 |
| **TC, mg/dL** | 195.36 ± 40.21 | 192.64 ± 38.70 | 0.017 |
| **HDL-C, mg/dL** | 52.00 ± 15.54 | 50.69 ± 15.29 | 0.003 |
| **LDL-C, mg/dL** | 115.92 ± 34.16 | 116.00 ± 35.01 | 0.930 |
| **RC, mg/dL** | 26.66 ± 26.21 | 25.74 ± 24.41 | 0.216 |
| **Baseline CRP, mg/L** | 2.39 ± 5.68 | 2.86 ± 7.86 | 0.006 |
| **Baseline TG, mg/dL** | 137.30 ± 126.36 | 134.58 ± 107.89 | 0.443 |
| **Baseline FBG, mg/dL** | 108.61 ± 29.43 | 110.05 ± 35.94 | 0.100 |
| **Baseline CTI** | 4.74 ± 0.56 | 4.77 ± 0.60 | 0.055 |
| **Following CTI** | 4.83 ± 0.61 | 4.83 ± 0.61 | 0.886 |
| **Cumulative CTI** | 14.34 ± 1.55 | 14.40 ± 1.56 | 0.209 |
| **Age group, n (%)** |  |  | 0.494 |
| <60 | 777 (55.7%) | 9239 (56.7%) |  |
| >=60 | 617 (44.3%) | 7049 (43.3%) |  |
| **Sex, n (%)** |  |  | <0.001 |
| Female | 556 (40.0%) | 7915 (48.6%) |  |
| Male | 835 (60.0%) | 8386 (51.4%) |  |
| **Marital status, n (%)** |  |  | 0.011 |
| Unmarried | 147 (10.5%) | 2111 (13.0%) |  |
| Married | 1247 (89.5%) | 14170 (87.0%) |  |
| **Education level, n (%)** |  |  | <0.001 |
| < High school | 1314 (94.4%) | 14093 (86.7%) |  |
| >= High school | 78 (5.6%) | 2168 (13.3%) |  |
| **Residence place, n (%)** |  |  | <0.001 |
| Rural | 968 (69.4%) | 9569 (58.7%) |  |
| Urban | 426 (30.6%) | 6742 (41.3%) |  |
| **Geographic region, n (%)** |  |  | <0.001 |
| South | 906 (65.0%) | 8960 (54.9%) |  |
| North | 488 (35.0%) | 7351 (45.1%) |  |
| **Smoking status, n (%)** |  |  | <0.001 |
| Never | 920 (66.0%) | 9706 (60.1%) |  |
| Ever | 100 (7.2%) | 1969 (12.2%) |  |
| Now | 374 (26.8%) | 4488 (27.8%) |  |
| **Drinking status, n (%)** |  |  | 0.122 |
| Nondrinker | 919 (70.3%) | 10878 (72.3%) |  |
| Drinker | 389 (29.7%) | 4168 (27.7%) |  |
| **Social activities, n (%)** |  |  | 0.340 |
| No | 1173 (84.1%) | 13559 (83.1%) |  |
| Yes | 221 (15.9%) | 2755 (16.9%) |  |
| **Diabetes, n (%)** |  |  | 0.232 |
| No | 1304 (95.0%) | 15108 (94.2%) |  |
| Yes | 68 (5.0%) | 926 (5.8%) |  |
| **Cancer, n (%)** |  |  | 0.602 |
| No | 1371 (99.3%) | 15958 (99.1%) |  |
| Yes | 10 (0.7%) | 145 (0.9%) |  |
| **CLD, n (%)** |  |  | 0.063 |
| No | 1226 (88.1%) | 14454 (89.8%) |  |
| Yes | 165 (11.9%) | 1649 (10.2%) |  |
| **Memory-related disease, n (%)** |  |  | 0.007 |
| No | 1381 (99.3%) | 15836 (98.3%) |  |
| Yes | 10 (0.7%) | 278 (1.7%) |  |
| **Digestive system disease, n (%)** |  |  | <0.001 |
| No | 945 (67.9%) | 12545 (77.8%) |  |
| Yes | 446 (32.1%) | 3579 (22.2%) |  |

Abbreviations: CTI, C-reactive protein-triglyceride-glucose index; eGFR, estimated glomerular filtration rate; CLD, chronic lung disease; TC, total cholesterol; HDL-C, high-density lipoprotein cholesterol; LDL-C, low-density lipoprotein cholesterol; RC, remnant cholesterol; CRP, C-reactive protein; TG, triglyceride; FBG, fasting blood glucose. Digestive system disease in the main analyses was defined based on liver disease and stomach disease.

**Table S4.** Characteristics of included and excluded participants in the hospital-based cross-sectional replication cohort.

| Variable | Included (n=7960) | Excluded (n=10792) | *P*-value |
| --- | --- | --- | --- |
| **Age, years** | 62.17 ± 10.63 | 50.21 ± 18.59 | <0.001 |
| **Sleep duration, h** | 6.58 ± 1.41 | 6.60 ± 1.41 | 0.301 |
| **eGFR, ml/min/1.73 m²** | 82.35 ± 18.29 | 93.69 ± 23.74 | <0.001 |
| **TC, mg/dL** | 203.28 ± 34.35 | 201.73 ± 34.26 | 0.003 |
| **HDL-C, mg/dL** | 49.35 ± 11.62 | 49.67 ± 11.50 | 0.071 |
| **LDL-C, mg/dL** | 123.05 ± 28.94 | 122.94 ± 29.33 | 0.802 |
| **RC, mg/dL** | 30.88 ± 14.51 | 29.40 ± 14.24 | <0.001 |
| **Baseline CRP, mg/L** | 3.60 ± 4.29 | 3.19 ± 3.51 | <0.001 |
| **Baseline TG, mg/dL** | 159.38 ± 80.62 | 149.87 ± 76.47 | <0.001 |
| **Baseline FBG, mg/dL** | 107.72 ± 24.28 | 100.53 ± 24.82 | <0.001 |
| **Baseline CTI** | 5.17 ± 0.53 | 5.09 ± 0.53 | <0.001 |
| **Age group, n (%)** |  |  | <0.001 |
| <60 | 3366 (42.3%) | 6687 (62.9%) |  |
| >=60 | 4594 (57.7%) | 3949 (37.1%) |  |
| **Sex, n (%)** |  |  | 1.000 |
| Female | 2269 (28.5%) | 3076 (28.5%) |  |
| Male | 5691 (71.5%) | 7716 (71.5%) |  |
| **Marital status, n (%)** |  |  | <0.001 |
| Unmarried | 2059 (25.9%) | 4312 (42.5%) |  |
| Married | 5901 (74.1%) | 5824 (57.5%) |  |
| **Education level, n (%)** |  |  | <0.001 |
| < High school | 6260 (78.6%) | 7108 (70.9%) |  |
| >= High school | 1700 (21.4%) | 2916 (29.1%) |  |
| **Residence place, n (%)** |  |  | 0.083 |
| Rural | 2962 (37.6%) | 3765 (36.3%) |  |
| Urban | 4916 (62.4%) | 6596 (63.7%) |  |
| **Geographic region, n (%)** |  |  | 0.197 |
| South | 4208 (53.4%) | 5436 (52.4%) |  |
| North | 3673 (46.6%) | 4934 (47.6%) |  |
| **Smoking status, n (%)** |  |  | <0.001 |
| Never | 5557 (69.8%) | 7228 (72.6%) |  |
| Ever | 841 (10.6%) | 887 (8.9%) |  |
| Now | 1562 (19.6%) | 1836 (18.5%) |  |
| **Drinking status, n (%)** |  |  | <0.001 |
| Never | 5406 (67.9%) | 7052 (70.4%) |  |
| Ever | 796 (10.0%) | 890 (8.9%) |  |
| Now | 1758 (22.1%) | 2072 (20.7%) |  |
| **Social activities, n (%)** |  |  | <0.001 |
| No | 6011 (77.2%) | 7507 (73.2%) |  |
| Yes | 1775 (22.8%) | 2743 (26.8%) |  |
| **Diabetes, n (%)** |  |  | <0.001 |
| No | 6169 (77.5%) | 8791 (81.5%) |  |
| Yes | 1791 (22.5%) | 2001 (18.5%) |  |
| **Cancer, n (%)** |  |  | <0.001 |
| No | 7643 (96.0%) | 10505 (97.3%) |  |
| Yes | 317 (4.0%) | 287 (2.7%) |  |
| **CLD, n (%)** |  |  | <0.001 |
| No | 6287 (79.0%) | 8872 (82.2%) |  |
| Yes | 1673 (21.0%) | 1920 (17.8%) |  |
| **Memory-related disease, n (%)** |  |  | <0.001 |
| No | 7299 (91.7%) | 10153 (94.1%) |  |
| Yes | 661 (8.3%) | 639 (5.9%) |  |
| **Digestive system disease, n (%)** |  |  | 0.001 |
| No | 4310 (54.1%) | 6098 (56.5%) |  |
| Yes | 3650 (45.9%) | 4694 (43.5%) |  |

Abbreviations: CTI, C-reactive protein-triglyceride-glucose index; eGFR, estimated glomerular filtration rate; CLD, chronic lung disease; TC, total cholesterol; HDL-C, high-density lipoprotein cholesterol; LDL-C, low-density lipoprotein cholesterol; RC, remnant cholesterol; CRP, C-reactive protein; TG, triglyceride; FBG, fasting blood glucose. Digestive system disease in the main analyses was defined based on liver disease and stomach disease.

**Table S5.** Variance inflation factors (VIFs) for covariates in fully adjusted Cox models (Model 3).

| **Covariate** | **Baseline CTI** | **cumCTI** | **CTI change** |
| --- | --- | --- | --- |
| **Age** | 1.290 | 1.297 | 1.297 |
| **Digestive system disease** | 1.034 | 1.046 | 1.046 |
| **Diabetes** | 1.084 | 1.096 | 1.096 |
| **Cancer** | 1.005 | 1.003 | 1.003 |
| **Chronic lung disease** | 1.036 | 1.045 | 1.045 |
| **memory-related disease** | 1.017 | 1.023 | 1.023 |
| **Drinking status** | 1.362 | 1.398 | 1.398 |
| **Education level** | 1.091 | 1.096 | 1.096 |
| **eGFR** | 1.225 | 1.248 | 1.248 |
| **Marital status** | 1.062 | 1.063 | 1.063 |
| **Sleep duration** | 1.036 | 1.041 | 1.041 |
| **RC** | 1.100 | 1.120 | 1.120 |
| **Residence place** | 1.057 | 1.059 | 1.059 |
| **Sex** | 1.977 | 2.045 | 2.045 |
| **Smoking status** | 1.792 | 1.837 | 1.837 |
| **Social activities** | 1.027 | 1.026 | 1.026 |
| **Geographic region** | 1.044 | 1.042 | 1.042 |
| Notes: Values are variance inflation factors (VIF). For categorical covariates, GVIF was converted to GVIF^(1/(2*Df)). | | | |
| All VIFs were computed from Cox models including Model 3 covariates only; columns reflect analysis datasets for baseline CTI (n=2894), cumCTI (n=1394), and CTI change (n=1394). | | | |
| A commonly used threshold is 2.5, below which multicollinearity is not considered problematic. | | | |

**Table S6.** Baseline characteristics according to baseline CTI quartiles in the hospital-based cross-sectional replication cohort.

| **Baseline Characteristics by baseline CTI Quartiles** | | | | | | | |
| --- | --- | --- | --- | --- | --- | --- | --- |
| **Characteristic** | **Overall (n=7960)** | **Q1 (n=1865)** | **Q2 (n=1988)** | **Q3 (n=2041)** | **Q4 (n=2066)** | ***P*-value**^a^ | ***P*-adjust**^b^ |
| **Age, years** | 62.00 (54.00, 69.00) | 58.00 (51.00, 65.00) | 61.00 (54.00, 69.00) | 62.00 (55.00, 70.00) | 65.00 (57.00, 73.00) | <0.001 | <0.001 |
| **eGFR, ml/min/1.73 m²** | 82.97 (68.44, 97.47) | 85.12 (70.81, 99.79) | 84.00 (68.65, 98.57) | 82.03 (67.32, 96.12) | 81.82 (66.72, 95.32) | <0.001 | <0.001 |
| **RC, mg/dL** | 29.02 (20.85, 38.59) | 23.79 (16.66, 31.45) | 27.71 (19.98, 35.97) | 30.19 (22.53, 39.35) | 35.31 (26.13, 45.64) | <0.001 | <0.001 |
| **CRP, mg/L** | 2.39 (1.30, 4.34) | 0.92 (0.60, 1.33) | 1.80 (1.28, 2.54) | 3.00 (2.10, 4.18) | 6.00 (4.00, 9.02) | <0.001 | <0.001 |
| **TG, mg/dL** | 142.70 (103.40, 195.29) | 96.53 (73.28, 124.98) | 128.82 (101.37, 163.05) | 155.41 (121.13, 199.25) | 205.46 (160.72, 269.39) | <0.001 | <0.001 |
| **FBG, mg/dL** | 105.26 (90.29, 121.61) | 89.50 (78.55, 101.93) | 100.73 (88.53, 114.98) | 108.53 (95.90, 122.89) | 121.49 (106.76, 139.56) | <0.001 | <0.001 |
| **CTI** | 5.17 (0.53) | 4.48 (0.26) | 4.98 (0.10) | 5.32 (0.10) | 5.82 (0.27) | <0.001 | <0.001 |
| **Age group,**  **n (%)** |  |  |  |  |  | <0.001 | <0.001 |
| <60 | 3366 (42.3) | 1034 (55.4) | 879 (44.2) | 815 (39.9) | 638 (30.9) |  |  |
| >=60 | 4594 (57.7) | 831 (44.6) | 1109 (55.8) | 1226 (60.1) | 1428 (69.1) |  |  |
| **Sex, n (%)** |  |  |  |  |  | <0.001 | <0.001 |
| Female | 2269 (28.5) | 384 (20.6) | 498 (25.1) | 614 (30.1) | 773 (37.4) |  |  |
| Male | 5691 (71.5) | 1481 (79.4) | 1490 (74.9) | 1427 (69.9) | 1293 (62.6) |  |  |
| **Marital status, n (%)** |  |  |  |  |  | 0.466 | 0.492 |
| Unmarried | 2059 (25.9) | 458 (24.6) | 532 (26.8) | 531 (26.0) | 538 (26.0) |  |  |
| Married | 5901 (74.1) | 1407 (75.4) | 1456 (73.2) | 1510 (74.0) | 1528 (74.0) |  |  |
| **Education level, n (%)** |  |  |  |  |  | 0.952 | 0.952 |
| < High school | 6260 (78.6) | 1471 (78.9) | 1555 (78.2) | 1610 (78.9) | 1624 (78.6) |  |  |
| >= High school | 1700 (21.4) | 394 (21.1) | 433 (21.8) | 431 (21.1) | 442 (21.4) |  |  |
| **Smoking status, n (%)** |  |  |  |  |  | <0.001 | <0.001 |
| Never | 5557 (69.8) | 1409 (75.5) | 1465 (73.7) | 1366 (66.9) | 1317 (63.7) |  |  |
| Ever | 841 (10.6) | 171 (9.2) | 180 (9.1) | 231 (11.3) | 259 (12.5) |  |  |
| Current | 1562 (19.6) | 285 (15.3) | 343 (17.3) | 444 (21.8) | 490 (23.7) |  |  |
| **Drinking status, n (%)** |  |  |  |  |  | <0.001 | <0.001 |
| Never | 5406 (67.9) | 1378 (73.9) | 1374 (69.1) | 1381 (67.7) | 1273 (61.6) |  |  |
| Ever | 796 (10.0) | 176 (9.4) | 200 (10.1) | 182 (8.9) | 238 (11.5) |  |  |
| Current | 1758 (22.1) | 311 (16.7) | 414 (20.8) | 478 (23.4) | 555 (26.9) |  |  |
| **Diabetes,**  **n (%)** |  |  |  |  |  | <0.001 | <0.001 |
| No | 6169 (77.5) | 1590 (85.3) | 1588 (79.9) | 1555 (76.2) | 1436 (69.5) |  |  |
| Yes | 1791 (22.5) | 275 (14.7) | 400 (20.1) | 486 (23.8) | 630 (30.5) |  |  |
| **Cancer, n (%)** |  |  |  |  |  | 0.274 | 0.306 |
| No | 7643 (96.0) | 1796 (96.3) | 1918 (96.5) | 1959 (96.0) | 1970 (95.4) |  |  |
| Yes | 317 (4.0) | 69 (3.7) | 70 (3.5) | 82 (4.0) | 96 (4.6) |  |  |
| **CLD, n (%)** |  |  |  |  |  | 0.008 | 0.009 |
| No | 6287 (79.0) | 1522 (81.6) | 1573 (79.1) | 1585 (77.7) | 1607 (77.8) |  |  |
| Yes | 1673 (21.0) | 343 (18.4) | 415 (20.9) | 456 (22.3) | 459 (22.2) |  |  |
| **memory-related disease, n (%)** |  |  |  |  |  | <0.001 | <0.001 |
| No | 7299 (91.7) | 1749 (93.8) | 1831 (92.1) | 1877 (92.0) | 1842 (89.2) |  |  |
| Yes | 661 (8.3) | 116 (6.2) | 157 (7.9) | 164 (8.0) | 224 (10.8) |  |  |
| **Digestive system disease, n (%)** |  |  |  |  |  | <0.001 | <0.001 |
| No | 4310 (54.1) | 1085 (58.2) | 1072 (53.9) | 1085 (53.2) | 1068 (51.7) |  |  |
| Yes | 3650 (45.9) | 780 (41.8) | 916 (46.1) | 956 (46.8) | 998 (48.3) |  |  |
| **CVD, n (%)** |  |  |  |  |  | <0.001 | <0.001 |
| No | 4816 (60.5) | 1364 (73.1) | 1271 (63.9) | 1182 (57.9) | 999 (48.4) |  |  |
| Yes | 3144 (39.5) | 501 (26.9) | 717 (36.1) | 859 (42.1) | 1067 (51.6) |  |  |
| Abbreviations: CTI, C-reactive protein-triglyceride-glucose index; CVD, Cardiovascular Disease; eGFR, estimated glomerular filtration rate; CLD, chronic lung disease; RC, remnant cholesterol; CRP, C-reactive protein; TG, triglyceride; FBG, fasting blood glucose. | | | | | | | |
| ^a^: P-values for continuous variables calculated using ANOVA or Kruskal-Wallis test; for categorical variables using Chi-squared test. | | | | | | | |
| ^b^: Adjusted P-values calculated using the Benjamini-Hochberg method. | | | | | | | |

**Table S7.** Threshold effect analysis of baseline CTI with new-onset CVD.

| **Outcome: CVD** | **HR (95%CI)** | ***P*-value^a^** |
| --- | --- | --- |
| **Model I** |  |  |
| One line effect | 1.250 (1.081, 1.444) | **0.002** |
| **Model II** |  |  |
| Turning point (K) | 4.897 | |
| < K effect 1 | 1.577 (1.218, 2.042) | **<0.001** |
| > K effect 2 | 0.984 (0.754, 1.284) | **0.902** |
| LRT test | **0.029** | |
| Table data: HR (95%CI) *P*-value  Outcome variable: CVD  Exposure variable: baseline CTI  ^a^ Adjusted for: age, sex, marital status, education level, residence place, geographic region, smoking status, drinking status, sleep duration, social activities, diabetes, cancer, chronic lung disease, memory-related disease, digestive system disease, eGFR, and RC.  Abbreviations: CTI, C-reactive protein-triglyceride-glucose index; CVD, cardiovascular disease; HR, hazard ratio; CI, confidence interval; LRT, log-likelihood ratio test; eGFR, estimated glomerular filtration rate; RC, remnant cholesterol. | | |

**Table S8.** Incremental predictive performance for 9-year CVD in CHARLS arthritis cohort.

| **Model** | **Harrell's C index** | ***P*-value** | **Continuous NRI**  **(95% CI)** | ***P*-value** | **IDI (95% CI)** | ***P*-value** |
| --- | --- | --- | --- | --- | --- | --- |
| CVD | - | - | - | - | - | - |
| Basic model | 0.615 (0.594-0.636) | - | - | - | - | - |
| Basic model + CRP | 0.615 (0.594-0.636) | 0.902 | 0.073 (-0.004-0.154) | 0.064 | 0.000 (-0.000-0.000) | 0.351 |
| Basic model + FBG | 0.615 (0.594-0.636) | 0.776 | -0.005 (-0.103-0.080) | 0.913 | 0.000 (-0.000-0.000) | 0.411 |
| Basic model + TG | 0.615 (0.595-0.636) | 0.321 | -0.023 (-0.121-0.073) | 0.641 | 0.000 (-0.000-0.000) | 0.119 |
| Basic model + CTI | 0.618 (0.597-0.638) | 0.378 | 0.157 (0.059-0.257) | **0.002** | 0.003 (0.001-0.005) | **0.006** |
| Note: Basic models were adjusted for age group, sex, marital status, education level, residence place, geographic region, smoking status, drinking status, sleep duration, social activities, diabetes, cancer, chronic lung diseases, Memory-related disease, digestive system disease, eGFR, and RC. | | | | | | |
| Abbreviations: C, concordance; NRI, net reclassification improvement; IDI, integrated discrimination improvement; CI, confidence interval; CVD, cardiovascular disease; CTI, C-reactive protein-triglyceride-glucose index; CRP, C-reactive protein; FBG, fasting blood glucose; TG, triglyceride; RC, remnant cholesterol; eGFR, estimated glomerular filtration rate; IPCW, inverse probability of censoring weighting. | | | | | | |

**Table S9.** Sensitivity analysis excluding participants who developed CVD within the first 2 years of follow-up.

| **Characteristic** | **Model 1** | ***P*-value** | **Model 2** | ***P*-value** | **Model 3** | ***P*-value** |
| --- | --- | --- | --- | --- | --- | --- |
|  | **HR (95%CI)** |  | **HR (95%CI)** |  | **HR (95%CI)** |  |
| **CTI (per 1 unit increase)** | 1.262 (1.117, 1.427) | **<0.001** | 1.253 (1.107, 1.418) | **<0.001** | 1.262 (1.088, 1.465) | **0.002** |
| Quartile CTI |  |  |  |  |  |  |
| Q1 | Ref. |  | Ref. |  | Ref. |  |
| Q2 | 1.337 (1.074, 1.665) | **0.009** | 1.292 (1.037, 1.609) | **0.022** | 1.269 (1.017, 1.585) | **0.035** |
| Q3 | 1.562 (1.261, 1.935) | **<0.001** | 1.509 (1.218, 1.870) | **<0.001** | 1.470 (1.178, 1.834) | **<0.001** |
| Q4 | 1.611 (1.299, 1.996) | **<0.001** | 1.575 (1.271, 1.953) | **<0.001** | 1.577 (1.243, 2.002) | **<0.001** |
| *P* for trend |  | **<0.001** |  | **<0.001** |  | **<0.001** |
| **cumCTI (per 1 unit increase)** | 1.108 (1.032, 1.190) | **0.005** | 1.117 (1.040, 1.200) | **0.003** | 1.118 (1.025, 1.220) | **0.012** |
| Quartile cumCTI |  |  |  |  |  |  |
| Q1 | Ref. |  | Ref. |  | Ref. |  |
| Q2 | 1.084 (0.767, 1.532) | 0.647 | 1.088 (0.770, 1.538) | 0.632 | 1.082 (0.762, 1.535) | 0.661 |
| Q3 | 1.453 (1.046, 2.017) | **0.026** | 1.435 (1.033, 1.996) | **0.032** | 1.418 (1.009, 1.995) | **0.045** |
| Q4 | 1.503 (1.082, 2.089) | **0.015** | 1.537 (1.106, 2.137) | **0.010** | 1.504 (1.038, 2.179) | **0.031** |
| *P* for trend |  | **0.004** |  | **0.003** |  | **0.012** |
| **CTI change pattern** |  |  |  |  |  |  |
| Cluster 1 | Ref. |  | Ref. |  | Ref. |  |
| Cluster 2 | 1.401 (1.075, 1.826) | **0.013** | 1.410 (1.081, 1.839) | **0.011** | 1.402 (1.065, 1.844) | **0.016** |
| Cluster 3 | 1.469 (1.065, 2.026) | **0.019** | 1.511 (1.095, 2.085) | **0.012** | 1.443 (1.001, 2.081) | 0.050 |
| *P* for trend |  | **0.010** |  | **0.006** |  | **0.026** |
| Model 1: unadjusted for any covariates | | | | | | |
| Model 2: adjusted for age, sex and marital status | | | | | | |
| Model 3: adjusted for age, sex, marital status, education level, residence place, geographic region, smoking status, drinking status, sleep duration, social activities, diabetes, cancer, chronic lung disease, memory-related disease, digestive system disease, eGFR, and RC | | | | | | |
| Abbreviations: CTI, C-reactive protein-triglyceride-glucose index; CVD, cardiovascular disease; HR, hazard ratio; CI, confidence interval; Q, quartile; Ref, reference; eGFR, estimated glomerular filtration rate; RC, remnant cholesterol. | | | | | | |

**Table S10.** Sensitivity analysis including only participants with complete covariate data.

| **Characteristic** | **Model 1** | ***P*-value** | **Model 2** | ***P*-value** | **Model 3** | ***P*-value** |
| --- | --- | --- | --- | --- | --- | --- |
|  | **HR (95%CI)** |  | **HR (95%CI)** |  | **HR (95%CI)** |  |
| **CTI (per 1 unit increase)** | 1.304 (1.145, 1.486) | **<0.001** | 1.297 (1.137, 1.479) | **<0.001** | 1.268 (1.085, 1.483) | **0.003** |
| **Quartile CTI** |  |  |  |  |  |  |
| Q1 | Ref. |  | Ref. |  | Ref. |  |
| Q2 | 1.469 (1.169, 1.844) | **<0.001** | 1.419 (1.129, 1.783) | **0.003** | 1.380 (1.095, 1.738) | **0.006** |
| Q3 | 1.600 (1.278, 2.004) | **<0.001** | 1.557 (1.243, 1.950) | **<0.001** | 1.466 (1.161, 1.850) | **0.001** |
| Q4 | 1.674 (1.337, 2.096) | **<0.001** | 1.643 (1.311, 2.057) | **<0.001** | 1.572 (1.225, 2.018) | **<0.001** |
| *P* for trend |  | **<0.001** |  | **<0.001** |  | **<0.001** |
| **cumCTI (per 1 unit increase)** | 1.101 (1.018, 1.190) | **0.016** | 1.108 (1.024, 1.198) | **0.010** | 1.089 (0.991, 1.197) | 0.076 |
| **Quartile cumCTI** |  |  |  |  |  |  |
| Q1 | Ref. |  | Ref. |  | Ref. |  |
| Q2 | 1.031 (0.714, 1.489) | 0.869 | 1.039 (0.719, 1.501) | 0.838 | 1.000 (0.689, 1.452) | 0.999 |
| Q3 | 1.424 (1.007, 2.015) | **0.046** | 1.424 (1.005, 2.018) | **0.047** | 1.345 (0.937, 1.930) | 0.108 |
| Q4 | 1.423 (1.003, 2.019) | **0.048** | 1.458 (1.027, 2.070) | **0.035** | 1.350 (0.911, 2.003) | 0.135 |
| *P* for trend |  | **0.013** |  | **0.010** |  | 0.057 |
| **CTI change pattern** |  |  |  |  |  |  |
| Cluster 1 | Ref. |  | Ref. |  | Ref. |  |
| Cluster 2 | 1.380 (1.043, 1.826) | **0.024** | 1.394 (1.052, 1.846) | **0.021** | 1.340 (1.001, 1.793) | **0.049** |
| Cluster 3 | 1.372 (0.968, 1.944) | 0.076 | 1.408 (0.992, 1.998) | 0.055 | 1.286 (0.867, 1.908) | 0.210 |
| *P* for trend |  | **0.041** |  | **0.029** |  | 0.128 |
| Model 1: unadjusted for any covariates | | | | | | |
| Model 2: adjusted for age, sex and marital status | | | | | | |
| Model 3: adjusted for age, sex, marital status, education level, residence place, geographic region, smoking status, drinking status, sleep duration, social activities, diabetes, cancer, chronic lung disease, memory-related disease, digestive system disease, eGFR, and RC | | | | | | |
| Abbreviations: CTI, C-reactive protein-triglyceride-glucose index; CVD, cardiovascular disease; HR, hazard ratio; CI, confidence interval; Q, quartile; Ref, reference; eGFR, estimated glomerular filtration rate; RC, remnant cholesterol. | | | | | | |

**Table S11.** Sensitivity analysis excluding participants who died during follow-up.

| **Characteristic** | **Model 1** | ***P*-value** | **Model 2** | ***P*-value** | **Model 3** | ***P*-value** |
| --- | --- | --- | --- | --- | --- | --- |
|  | **HR (95%CI)** |  | **HR (95%CI)** |  | **HR (95%CI)** |  |
| **CTI (per 1 unit increase)** | 1.293 (1.145, 1.461) | **<0.001** | 1.287 (1.138, 1.455) | **<0.001** | 1.287 (1.109, 1.493) | **<0.001** |
| **Quartile CTI** |  |  |  |  |  |  |
| Q1 | Ref. |  | Ref. |  | Ref. |  |
| Q2 | 1.409 (1.131, 1.756) | **0.002** | 1.358 (1.089, 1.692) | **0.007** | 1.332 (1.066, 1.664) | **0.012** |
| Q3 | 1.644 (1.327, 2.037) | **<0.001** | 1.591 (1.283, 1.973) | **<0.001** | 1.524 (1.221, 1.902) | **<0.001** |
| Q4 | 1.692 (1.365, 2.097) | **<0.001** | 1.658 (1.337, 2.055) | **<0.001** | 1.636 (1.288, 2.077) | **<0.001** |
| *P* for trend |  | **<0.001** |  | **<0.001** |  | **<0.001** |
| **cumCTI (per 1 unit increase)** | 1.122 (1.044, 1.207) | **0.002** | 1.131 (1.052, 1.217) | **<0.001** | 1.134 (1.037, 1.239) | **0.006** |
| **Quartile cumCTI** |  |  |  |  |  |  |
| Q1 | Ref. |  | Ref. |  | Ref. |  |
| Q2 | 1.069 (0.750, 1.523) | 0.712 | 1.075 (0.755, 1.532) | 0.688 | 1.065 (0.744, 1.524) | 0.731 |
| Q3 | 1.492 (1.069, 2.082) | **0.019** | 1.477 (1.057, 2.063) | **0.022** | 1.450 (1.026, 2.048) | **0.035** |
| Q4 | 1.551 (1.113, 2.162) | **0.010** | 1.585 (1.137, 2.211) | **0.007** | 1.550 (1.066, 2.252) | **0.022** |
| *P* for trend |  | **0.002** |  | **0.001** |  | **0.007** |
| **CTI change pattern** |  |  |  |  |  |  |
| Cluster 1 | Ref. |  | Ref. |  | Ref. |  |
| Cluster 2 | 1.399 (1.068, 1.831) | **0.015** | 1.403 (1.071, 1.839) | **0.014** | 1.394 (1.055, 1.843) | **0.020** |
| Cluster 3 | 1.521 (1.101, 2.103) | **0.011** | 1.562 (1.129, 2.160) | **0.007** | 1.487 (1.028, 2.151) | **0.035** |
| *P* for trend |  | **0.007** |  | **0.004** |  | **0.020** |
| Model 1: unadjusted for any covariates | | | | | | |
| Model 2: adjusted for age, sex and marital status | | | | | | |
| Model 3: adjusted for age, sex, marital status, education level, residence place, geographic region, smoking status, drinking status, sleep duration, social activities, diabetes, cancer, chronic lung disease, memory-related disease, digestive system disease, eGFR, and RC | | | | | | |
| Abbreviations: CTI, C-reactive protein-triglyceride-glucose index; CVD, cardiovascular disease; HR, hazard ratio; CI, confidence interval; Q, quartile; Ref, reference; eGFR, estimated glomerular filtration rate; RC, remnant cholesterol. | | | | | | |

**Table S12.** Sensitivity analysis further adjusted for BMI and physical activity.

| **Characteristic** | **Model 1** | ***P*-value** | **Model 2** | ***P*-value** | **Model 3** | ***P*-value** |
| --- | --- | --- | --- | --- | --- | --- |
|  | **HR (95%CI)** |  | **HR (95%CI)** |  | **HR (95%CI)** |  |
| **CTI (per 1 unit increase)** | 1.207  (1.040, 1.401) | **0.013** | 1.198  (0.930, 1.544) | 0.161 | 1.181  (0.910, 1.532) | 0.212 |
| **Quartile CTI** |  |  |  |  |  |  |
| Q1 | Ref. |  | Ref. |  | Ref. |  |
| Q2 | 1.283  (1.033, 1.594) | **0.024** | 1.287  (0.889, 1.863) | 0.181 | 1.279  (0.882, 1.855) | 0.194 |
| Q3 | 1.411  (1.133, 1.758) | **0.002** | 1.335  (0.918, 1.942) | 0.131 | 1.326  (0.909, 1.933) | 0.143 |
| Q4 | 1.505  (1.186, 1.909) | **<0.001** | 1.599  (1.072, 2.385) | **0.021** | 1.577  (1.049, 2.371) | **0.029** |
| ***P* for trend** |  | **<0.001** |  | **0.027** |  | **0.036** |
| **cumCTI (per 1 unit increase)** | 1.094  (0.999, 1.198) | 0.053 | 1.164  (1.003, 1.350) | **0.046** | 1.162  (0.997, 1.356) | 0.055 |
| **Quartile cumCTI** |  |  |  |  |  |  |
| Q1 | Ref. |  | Ref. |  | Ref. |  |
| Q2 | 1.040  (0.731, 1.479) | 0.829 | 0.697  (0.356, 1.364) | 0.292 | 0.683  (0.346, 1.349) | 0.272 |
| Q3 | 1.335  (0.943, 1.889) | 0.103 | 1.226  (0.681, 2.205) | 0.497 | 1.207  (0.667, 2.186) | 0.534 |
| Q4 | 1.360  (0.925, 2.000) | 0.118 | 1.453  (0.772, 2.736) | 0.247 | 1.402  (0.723, 2.718) | 0.317 |
| ***P* for trend** |  | 0.056 |  | 0.116 |  | 0.139 |
| **CTI change pattern** |  |  |  |  |  |  |
| Cluster 1 | Ref. |  | Ref. |  | Ref. |  |
| Cluster 2 | 1.322  (0.997, 1.752) | 0.052 | 1.212  (0.731, 2.009) | 0.457 | 1.207  (0.725, 2.010) | 0.469 |
| Cluster 3 | 1.304  (0.890, 1.909) | 0.173 | 1.706  (0.914, 3.183) | 0.093 | 1.688  (0.882, 3.230) | 0.114 |
| ***P* for trend** |  | 0.118 |  | 0.101 |  | 0.123 |
| Model 1: unadjusted for any covariates | | | | | | |
| Model 2: adjusted for age, sex and marital status | | | | | | |
| Model 3: adjusted for age, sex, marital status, education level, residence place, geographic region, smoking status, drinking status, sleep duration, social activities, diabetes, cancer, chronic lung disease, memory-related disease, digestive system disease, eGFR, and RC | | | | | | |
| Abbreviations: CTI, C-reactive protein-triglyceride-glucose index; CVD, cardiovascular disease; HR, hazard ratio; CI, confidence interval; Q, quartile; Ref, reference; eGFR, estimated glomerular filtration rate; RC, remnant cholesterol; BMI, body mass index; PA, physical activity. | | | | | | |

**Table S13.** Sensitivity analysis jointly adjusted for baseline CTI and delta CTI.

| **Characteristic** | **Model 1** | ***P*-value** | **Model 2** | ***P*-value** | **Model 3** | ***P*-value** |
| --- | --- | --- | --- | --- | --- | --- |
|  | **HR (95%CI)** |  | **HR (95%CI)** |  | **HR (95%CI)** |  |
| **Joint model of CTI in 2011 and delta CTI** |  |  |  |  |  |  |
| CTI in 2011 (per 1 unit increase) | 1.370  (1.106, 1.698) | **0.004** | 1.346  (1.083, 1.674) | **0.007** | 1.376  (1.056, 1.791) | **0.018** |
| Delta CTI (per 1 unit increase) | 1.083  (0.876, 1.337) | 0.462 | 1.076  (0.869, 1.333) | 0.501 | 1.082  (0.866, 1.351) | 0.488 |
| Model 1: unadjusted for any covariates | | | | | | |
| Model 2: adjusted for age, sex and marital status | | | | | | |
| Model 3: adjusted for age, sex, marital status, education level, residence place, geographic region, smoking status, drinking status, sleep duration, social activities, diabetes, cancer, chronic lung disease, memory-related disease, digestive system disease, eGFR, and RC | | | | | | |
| Abbreviations: CTI, C-reactive protein-triglyceride-glucose index; HR, hazard ratio; CI, confidence interval. | | | | | | |
